# A logic-incorporated gene regulatory network deciphers principles in cell fate decisions

**DOI:** 10.7554/eLife.88742

**Published:** 2024-04-23

**Authors:** Gang Xue, Xiaoyi Zhang, Wanqi Li, Lu Zhang, Zongxu Zhang, Xiaolin Zhou, Di Zhang, Lei Zhang, Zhiyuan Li

**Affiliations:** 1 Peking-Tsinghua Center for Life Sciences, Academy for Advanced Interdisciplinary Studies, Peking University Beijing China; 2 Center for Quantitative Biology, Academy for Advanced Interdisciplinary Studies, Peking University Beijing China; 3 Beijing International Center for Mathematical Research, Center for Machine Learning Research, Peking University Beijing China; Universidad Nacional Autónoma de México Mexico; École Normale Supérieure - PSL France

**Keywords:** gene regulatory network, cell fate decision, gene regulatory logic, driving force, gene expression noise, cell dynamics, Human, Mouse

## Abstract

Organisms utilize gene regulatory networks (GRN) to make fate decisions, but the regulatory mechanisms of transcription factors (TF) in GRNs are exceedingly intricate. A longstanding question in this field is how these tangled interactions synergistically contribute to decision-making procedures. To comprehensively understand the role of regulatory logic in cell fate decisions, we constructed a logic-incorporated GRN model and examined its behavior under two distinct driving forces (noise-driven and signal-driven). Under the noise-driven mode, we distilled the relationship among fate bias, regulatory logic, and noise profile. Under the signal-driven mode, we bridged regulatory logic and progression-accuracy trade-off, and uncovered distinctive trajectories of reprogramming influenced by logic motifs. In differentiation, we characterized a special logic-dependent priming stage by the solution landscape. Finally, we applied our findings to decipher three biological instances: hematopoiesis, embryogenesis, and trans-differentiation. Orthogonal to the classical analysis of expression profile, we harnessed noise patterns to construct the GRN corresponding to fate transition. Our work presents a generalizable framework for top-down fate-decision studies and a practical approach to the taxonomy of cell fate decisions.

## Introduction

**Figure 1.**
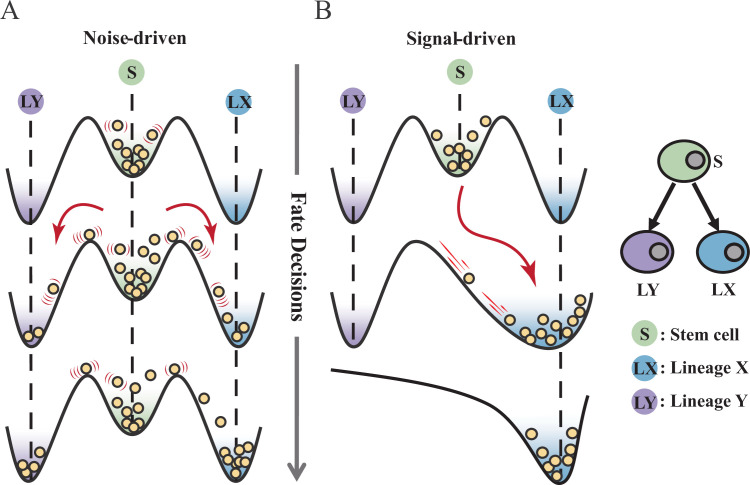
Schematic representation of cell fate decisions driven by noise (**A**) and signal (**B**) from a view of epigenetic landscape. (**A–B**) Valleys represent stable attractors. Cells (yellow balls) in stem cell fate (denoted as ‘S’, green well in landscape) differentiate into downstream fates, lineage X (denoted as ‘LX’, blue well), and lineage Y (denoted as ‘LY’, purple well). These abbreviations were used for following Figures 2—7.

Waddington’s epigenetic landscape is a fundamental and profound conceptualization of cell fate decisions (Waddington, 1957). Over decades, this insightful metaphor has facilitated researchers to distill a myriad of models regarding cell fate decisions (MacArthur, 2022; Schiebinger et al., 2019; Olsson et al., 2016; Bhattacharya et al., 2011; Fisher and Merkenschlager, 2010; Hota et al., 2022; Huang, 2012; Li et al., 2021). While introducing various quantitative models and dissecting diverse fate-decision processes, researchers have further elaborated the Waddington landscape (Shakiba et al., 2022; Loeffler and Schroeder, 2019; Coomer et al., 2022; Shi et al., 2022; Stanoev and Koseska, 2022; Glauche and Marr, 2021). An outstanding question is whether the landscape is static or not, i.e., whether cell fate decisions are driven by noise or signal (Stanoev and Koseska, 2022; Simon et al., 2018; Zernicka-Goetz and Huang, 2010). On one hand, some perspectives hold that cells reside in a stationary landscape, where decisions are made by switching through discrete valleys (Lord et al., 2019; Desai et al., 2021), as a result of gene expression noise (Chang et al., 2008; Guillemin and Stumpf, 2021) (termed as ‘noise-driven’; Figure 1A). Meanwhile, some researchers argued that the epigenetic landscape is dynamic during fate decisions. That is, the distortion of the landscape orchestrates fate transitions (Hota et al., 2022; Zhu et al., 2022; Li et al., 2020) and is driven by extrinsic signals (termed as ‘signal-driven’; Figure 1B).

Under the noise-driven mode, the bias of cell fate decisions largely depends on the spontaneous heterogeneity of gene expressions in the cell population (Wheat et al., 2020; Kovary et al., 2018). Consequently, the initial cellular state predominantly impacts the direction of the fate decision. Chang et al., 2008 uncovered that hematopoietic stem cell (HSC) population possesses intrinsic and robust heterogeneity of *Scal-1* expression (also known as *Ly6a* [van de Rijn et al., 1989]). Notably, populations with discrete expression levels of *Scal-1* confer different propensities for downstream lineage commitment. Considering the signal-driven mode, cell fates are tightly steered by extrinsic signals (e.g., cytokines, chemical molecules, mechanical strength, and genetic operations) that reshape the landscape (Figure 1B). In this circumstance, the impact of the initial state on fate decisions is relevantly inconsequential. Additionally, due to the accessibility of signal manipulation, the signal-driven mode has been widely utilized for cell fate engineering (Xu et al., 2015; Del Vecchio et al., 2017), leading to in-vitro induction systems centered on induced pluripotent stem cells (iPSC) for obtaining desired cell types (Ng et al., 2021). Recently, researchers reported a ‘fate-decision abduction’ of erythroid-to-myeloid trans-differentiation induced by various types of cancer, which facilitates tumor escape from the individual’s immune system (Long et al., 2022). Collectively, driving forces couple the foundational and crucial features of fate decisions, serving as an essential basis for further decoding fate decisions and interpreting the development of organisms (Simon et al., 2018). By examining the two driving modes, we can gain a better understanding and characterization of cell fate decisions, including in-vivo cell differentiation, oncogenesis, and in-vitro reprogramming systems.

Nevertheless, the driving forces that underlie fate decisions remain largely elusive. The intricate nature of GRNs presents a challenge in deciphering driving modes. It has been generally acknowledged that corresponding core GRNs orchestrate cell fate decisions (Qiu et al., 2022; Graf and Enver, 2009), where the lineage-specifying TFs interact to implement fate-decision procedures. Furthermore, researchers transferred specific TFs into donor cells to reconfigure the intracellular GRNs for acquiring cell types of interest (Hammelman et al., 2022; Mazid et al., 2022). Although some studies suggested that perturbation of a single TF is sufficient to transform certain cell fates (Ng et al., 2021), a large number of TFs are inevitably involved in most differentiation/reprogramming processes (Hersbach et al., 2022). In particular to orchestrate decisions among multiple cell fates, it is necessary for TFs to regulate target genes cooperatively (Trojanowski and Rippe, 2022). As crucial determinants of cell fates, TFs function via binding to cis-regulatory elements (CRE, e.g., promoter and enhancer). CREs of a single gene in metazoans can simultaneously accommodate numerous TFs (Lambert et al., 2018; Reiter et al., 2017). While experimental protocols have been developed to assess TF binding and one-to-one up- or down-regulatory relationships, it is more challenging to quantify these combinatorial regulations. For instance, given two factors activate and inhibit the same target gene, respectively, does the target gene turn on or off when both factors are present in its CREs?

Computational approaches in systems biology can be utilized to tackle complex networks (Macarthur et al., 2009; Krumsiek et al., 2011; Collombet et al., 2017; Moignard et al., 2015; Kirouac et al., 2009). A concise GRN model typically entails the following two elements. The element 1 is the topology. Much research efforts have been devoted to investigating network topologies on cellular behaviors, e.g., toggle switch (Graf and Enver, 2009; Davies et al., 2021), and feed-forward loop (Kittisopikul and Süel, 2010; Oliver Metzig et al., 2020; Sciammas et al., 2011). In particular, the Cross-Inhibition with Self-activation (CIS) network is one of the most studied two-node GRNs in cell fate decisions (Orkin and Zon, 2008; Kato and Igarashi, 2019), with examples found in *Gata1-PU.1* and *FLI1-KLF1* in hematopoiesis (Hoppe et al., 2016; Palii et al., 2019), *Nanog-Gata6* and *Oct4-Cdx2* in gastrulation (Simon et al., 2018; Zhou and Huang, 2011), and *Sir2* and *HAP* in yeast aging (Li et al., 2020). In this topology, two lineage-specifying factors inhibit each other while active themselves. For example, in the well-known *Gata1-PU.1* circuit, *Gata1* directs fate of megakaryocyte-erythroid progenitor (MEP), and *PU.1* (also known as *SPI1* in humans) specifies the fate of granulocyte-monocyte progenitor (GMP) (Orkin and Zon, 2008). Namely, the antagonism of two TFs implicates two cell fates in competition with each other.

The another element is the logic for regulatory functions (Ocone et al., 2015; Matsuura et al., 2018). Exemplified by the CIS network, each node (e.g., X and Y) receives the activation by itself and inhibition by the counterpart. Hence there is naturally the logic function between these two inputs. Given the logic function is AND, in the context of biological mechanism of regulation by TFs, *X* gene expresses only when X itself is present in X’s CREs but Y is not. Researchers observed in the *E. coli* lac operon system that changes in one single base can shift the regulatory logic significantly, suggesting that logic functions of GRNs can be adapted on the demand of specific functions in organisms (Collombet et al., 2017; Mayo et al., 2006; Buchler et al., 2003). Additionally, considering that the combination and cooperativity of TFs are of great significance in development (Zaret, 2020; Spitz and Furlong, 2012; Balsalobre and Drouin, 2022), theoretical investigation of the logic underlying GRNs should be concerned in cell fate decisions. However, despite the existence of large number of mathematical models on fate decisions, the role played by the regulatory logic in cell fate decisions is still obscure. Some theoretical studies put emphasis on specific biological instances, adopting logic functions that best fit the observations derived from experiments (Hota et al., 2022; Krumsiek et al., 2011; Huang et al., 2007). As a result, the models incorporated different regulatory logic received limited attention. Other research assigned logic to large-scale multi-node GRNs, confining the interpretation of the role of logic (Moignard et al., 2015; Wu et al., 2008). Collectively, the bridge between the logic of nodes in GRNs and cell fate decisions has not yet been elucidated systematically and adequately. Current research already encompassed a wealth of cell fate decisions: embryogenesis (Xu et al., 2022; Yu et al., 2022; Simunovic et al., 2022), lineage commitment (Palii et al., 2019; Pei et al., 2020; Psaila et al., 2020), oncogenesis (Aguadé-Gorgorió et al., 2021; Yang et al., 2022; Uthamacumaran, 2021), in-vitro reprogramming (Tang et al., 2022; Liang et al., 2019; Yu et al., 2021), and large-scale perturbations (Ng et al., 2021; Dixit et al., 2016; Joung et al., 2023; Replogle et al., 2022). Analogous to the effort on taxonomy of cell types and tumors (Domcke and Shendure, 2023), how cell fate decisions can be classified and distilled to the common properties is a challenge for further exploring systematically and application on fate engineering (MacArthur, 2023; Casey et al., 2020).

In this work, we integrated the fate-decision modes (noise-driven/signal-driven) and the classical logic operations (AND/OR) underlying GRNs in a continuous model. Based on our model, we investigated the impact of distinct logic operations on the nature of fate decisions with driving modes in consideration. Additionally, we extracted the difference in properties between the two driving modes. We unearthed that in the noise-driven stem cells, regulatory logic results in the opposite bias of fate decisions. We further distilled the relationship among noise profiles, logic motifs, and fate-decision bias, showing that knowledge of two of these allows inference of the third heuristically. Under the signal-driven mode, we identified two basic patterns of cell fate decisions: progression and accuracy. Moreover, based on our findings in silico, we characterized cell fate decisions in hematopoiesis and embryogenesis and unveiled their decision modes and logic motifs underlying GRNs. Ultimately, we applied our framework to a reprogramming system. We deciphered the driving force of this trans-differentiation, and utilized noise patterns for nominating key regulators. We underscored that clustering of gene noise patterns is an informative approach to investigate high-throughput datasets. Together, we underlined regulatory logic is of the significance in cell fate decisions. Our work presents a generalizable framework for classifying cell fate decisions and a blueprint for circuit design in synthetic biology.

## Results

### Section 1: Mathematical model of the CIS network with logic motifs

Binary tree-like cell fate decisions are prevalent in biological systems (Zhou and Huang, 2011; Domcke and Shendure, 2023; Stadler et al., 2021; Macnair et al., 2019), orchestrated by a series of the CIS networks. Accordingly, we developed our ordinary differential equations (ODE) model based on this paradigmatic and representative topology (Equations 1 and 2; see ‘Materials and methods’ for details).

(1) $$\frac{d\left[X\right]}{dt}=\frac{r_{0}^{x}+r_{1}k_{1}{\left[X\right]}^{n_{1}}+r_{2}k_{2}{\left[Y\right]}^{n_{2}}+r_{3}k_{3}{\left[X\right]}^{n_{1}}{\left[Y\right]}^{n_{2}}}{1+k_{1}{\left[X\right]}^{n_{1}}+k_{2}{\left[Y\right]}^{n_{2}}+k_{3}{\left[X\right]}^{n_{1}}{\left[Y\right]}^{n_{2}}}-d_{1}\left[X\right]$$

(2) $$\frac{d\left[Y\right]}{dt}=\frac{r_{0}^{y}+r_{4}k_{4}{\left[Y\right]}^{n_{2}}+r_{5}k_{5}{\left[X\right]}^{n_{1}}+r_{6}k_{6}{\left[X\right]}^{n_{1}}{\left[Y\right]}^{n_{2}}}{1+k_{4}{\left[Y\right]}^{n_{2}}+k_{5}{\left[X\right]}^{n_{1}}+k_{6}{\left[X\right]}^{n_{1}}{\left[Y\right]}^{n_{2}}}-d_{2}\left[Y\right]$$

**Figure 2.**
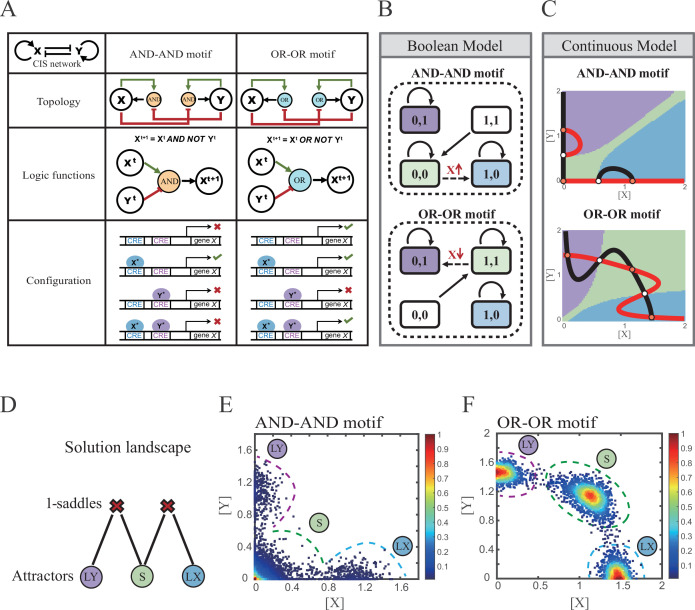
Models of the cross-inhibition with self-activation (CIS) network incorporated logic motifs. (**A**) A table listing the topologies with logic nodes, logic functions and Cis-regulatory elements (CRE) configurations in the CIS network incorporated AND-AND and OR-OR logic (denoted as AND-AND motif and OR-OR motif). X and Y are lineage-specifying transcription factors (TF). X^t+1^ indicates the value of X at the next time step. X^*^, Y^*^ represent activated forms of X and Y, respectively. The true or false signs denote whether gene *X* can be transcribed, respectively. These annotations were used for the following Figures 3—7. (**B**) State spaces of the AND-AND (top panel) and OR-OR (bottom panel) motifs in Boolean models. Updated rules of Boolean models are stated in Figure 2A. Rectangles indicate cell states. Green, blue, and purple represent S, LX, and LY, respectively. Solid arrows indicate transitions between states under corresponding Boolean models. Dotted arrows indicate forced transition imposed by external perturbations. (**C**) State spaces of the AND-AND (top panel) and OR-OR (bottom panel) motifs in ODE models. Dark and red lines represent nullclines of $\frac{d\left[X\right]}{dt}=0$, $\frac{d\left[Y\right]}{dt}=0$, respectively. Stable steady states (SSS) are denoted as orange dots. Unstable steady states (USS) are denoted as white dots. Each axis represents the concentration of each transcription factor, which units are arbitrary. Blue, green, and purple areas in state spaces indicate attractor basins representing LX, S, and LY, respectively. Color of each point in state space was assigned by the attractors they finally enter according to the deterministic models (Equation 1, Equation 2). These annotations were used for the following Figures 3—7. (**D**) The solution landscape both for the AND-AND and OR-OR motifs. The crimson X-cross sign denotes the first-order saddle node. Blue, green, and purple circles indicate attractors. These annotations were used for the following Figures 3—7. (**E–F**) Simulation result of stochastic differential equation models of the AND-AND (**E**) and OR-OR (**F**) motifs. Other than adding a white noise, parameters were identical with those in (**C**). Initial values were set to the attractor representing S fate in Figure 2C top panel (**E**) and Figure 2C bottom panel (**F**). Noise levels of *X* (*σ_x_*) and *Y* (*σ_y_*) are both set to 0.14 in the AND-AND motif (**E**), and 0.1 in the OR-OR motif (**F**). Stochastic simulation was preformed 3500 times, with each final state recorded as a dot on the plot. Color of heatmap corresponds to the density of points. Unit of concentration is arbitrary.

X and Y are TFs in the CIS network. *n_1_* and *n_2_* are the coefficients of molecular cooperation. *k_1_-k_3_* in Equation 1 and *k*_*4*_*-k*_*6*_ in Equation 2 represent the relative probabilities for possible configurations of binding of TFs and CREs. (Figure 2A). *d_1_* and *d_2_* are degradation rates of X and Y, respectively. Here, we considered a total of four CRE’s configurations as shown in Figure 2A (i.e., TFs bind to the corresponding CREs or not, 2^2^=4). Accordingly, depending on the transcription rates (i.e., *r_0_^x^*, *r_1_*, *r_2_*, *r_3_* in Equation 1, similarly in Equation 2) of each configuration, we can model the dynamics of TFs in the Shea-Ackers formalism (Shea and Ackers, 1985; Olariu and Peterson, 2019).

Thus, the distinct logic operations (AND/OR) of two inputs (e.g., activation by X itself and inhibition by Y) can be further implemented by assigning the corresponding profile of transcription rates in four configurations (Figure 2A). From the perspective of molecular biology, the regulatory logics embody the complicated nature of TF regulation that TFs function in a context-dependent manner. Considering the CIS network, when X and Y bind respective CREs concurrently, whether the expression of target gene is turned on or off depends on the different regulatory logics (specifically, off in the AND logic and on in the OR logic; Figure 2A). Notably, instead of exploring the different logics of one certain gene (Kittisopikul and Süel, 2010), we focus on different combinations of regulatory logics due to dynamics in cell fate decisions is generally orchestrated by GRNs with multiple TFs.

Benchmarking the Boolean models with different logic motifs (Figure 2B; see ‘Materials and methods’), we reproduced the geometry of the attractor basin in the continuous models resembling those represented by corresponding Boolean models (Figure 2C; see ‘Materials and methods’). Under double AND and double OR motifs (termed as AND-AND motif and OR-OR motif, respectively), there are typically three stable steady states (SSS) in the state spaces (Figure 2C): two attractors near the axes representing the fate of lineage X (denoted as LX, *X*^high^*Y*^low^) and the fate of lineage Y (denoted as LY, *X*^low^*Y*^high^), and the attractor in the center of the state space representing stem cell fate (denoted as S).

Evidently, the stem cell states exhibit different expression patterns between the two logic motifs. Stem cells in the AND-AND motif do not express *X* nor *Y* (Figure 2B top panel; expressed in low level in Figure 2C top panel), while in the OR-OR motif, stem cells express both lineage-specifying TFs (Figure 2B bottom panel; expressed in high level in Figure 2C bottom panel). The difference in the status of S attractors relates to the co-expression level of lineage-specifying TFs in stem cells in real biological systems (Loeffler and Schroeder, 2019; Palii et al., 2019). Intuitively, from the view of the Boolean model, stem cell state in the AND-AND motif ([0,0] state) needs to switch on lineage-specifying TFs to transit to downstream fates (Figure 2B top panel). Whereas in the OR-OR motif, fate transitions are subject to the switch-off of TF expression (Figure 2B bottom panel). Furthermore, we introduced the solution landscape method. Solution landscape is a pathway map consisting of all stationary points and their connections, which can describe different cell states and transfer paths of them (Yin et al., 2020; Yin et al., 2021). From the perspective of the solution landscape, two logic motifs possess akin geometric topologies in their steady-state adjacencies (Figure 2D): when there are three fates coexisting in the state space, S attractor resides in the middle of LX and LY as the possible pivot for fate transitions (Figure 2D). To investigate noise, we developed models with stochastic forms (see ‘Materials and methods’). Simulations display the primary distribution of cell populations, corresponding to SSSs in deterministic models (Figure 2E and F).

### Section 2: Two logic motifs exhibit opposite bias of fate decisions under the noise-driven mode

**Figure 3.**
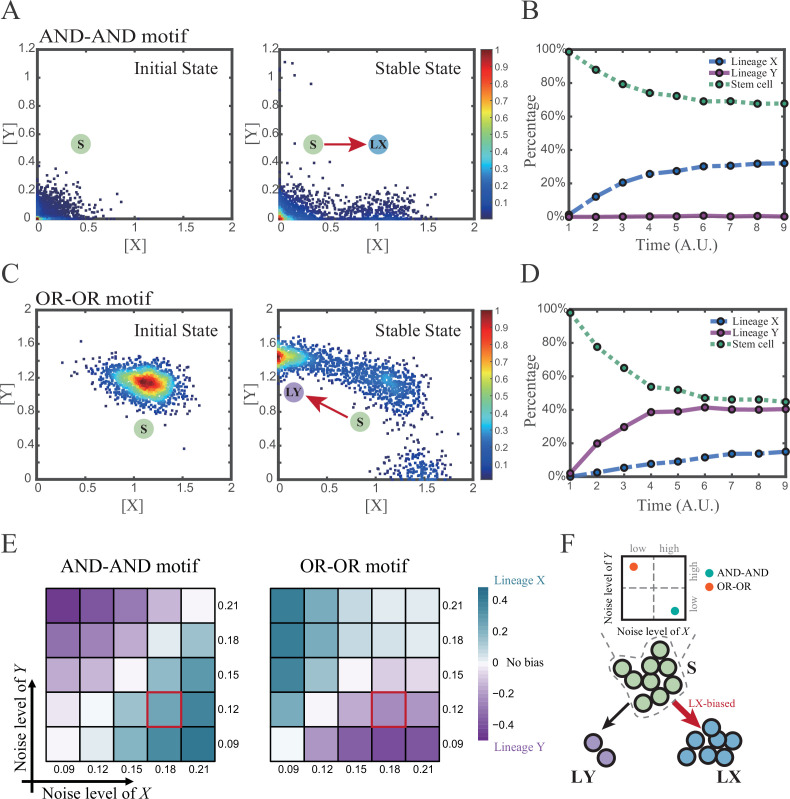
Two logic motifs exhibit opposite bias of fate decisions under the noise-driven mode. (**A and C**) Stochastic simulation in both the AND-AND and OR-OR motifs. *σ_x_* is set to 0.18, and *σ*_y_ is 0.12. In both (**A**) and (**C**), initial values were identical with attractors of stem cell fate in Figure 2C (SSSs in green attractor basins). Simulation was preformed 1500 times, with each initial (A left and C left) and final (A right and C right) states recorded as a dot on the plot. (**B and D**) Time courses of the percentage of cells in different fates in stochastic simulation, under the AND-AND motif (**B**) and OR-OR motif (**D**). Fates of cells were assigned by their final states according to the basins of the deterministic models in Figure 2C. Unit of time is arbitrary. (**E**) Heatmaps showing the bias of cell fate decisions under different noise levels of *X* and *Y*. Color of heatmap indicates the extent of bias. Here, $bias=\frac{n_{LX}-n_{LY}}{n_{total}}$ . *n_LX_*, *n_LY_* represent number of LX, LY, respectively. *n_total_* represents the total number of cells (n_total_=1500). The method of assigning fate to cells is identical with Figure 3B and D. The red-marked cells correspond to the noise conditions simulated in (**A**) and (**C**). (**F**) Schematic illustration in that stem cell populations possessing the same bias of fate decisions need to have opposite noise patterns, according to whether they are in the AND-AND or OR-OR motif. The red and bold arrow indicates the bias of fate decisions.

We first investigated the difference between the AND-AND and OR-OR motifs under the noise-driven mode. Here, we assigned the stem cell state as the starting point in simulation. In biological systems, it is unlikely that the noise level of different genes is kept perfectly the same. Asymmetry of the noise levels was thus introduced. First, we set the noise level of TF *X* higher than that of *Y* (*σ_x_*=0.18, *σ_y_*=0.12). Under this asymmetric noise, we observed that stem cells shifted toward LX in the AND-AND motif (Figure 3A and B), but toward LY in the OR-OR motif (Figure 3C and D). From the perspective of the state space, such properties intuitively originate from the distinctive status of stem cell attractors in two logic motifs (Figure 2B and C). In the AND-AND motif, the stem cell state resides at the origin of coordinates. Thus, with increasing *X*’s noise level, the stem cell population crossed the boundary between S and LX basins with a rising probability (Figure 2C top panel). Consequently, the fate decision of the stem cell population manifests a bias toward LX. Likewise, in the OR-OR motif, the stem cell population has a higher probability of entering LY basin following an increase in *X*’s noise (Figure 2C bottom panel). Next, we simulated multiple sets of noise levels for *X* and *Y*. We quantified the distribution of cell types, which was determined by the basin in which the final state of each round of stochastic simulation ended up. We observed that stem cell population displays almost opposite differentiation preference under identical noise levels but distinct logic motifs (Figure 3E). Conversely, when two distinct logic motifs exhibiting the same fate-decision bias, cell populations need to employ opposite noise patterns (Figure 3E and F). Collectively, if two of the three (noise profile, logic motif, fate-decision bias) are accessible, the last is inferential.

**Figure 3—figure supplement 1.**
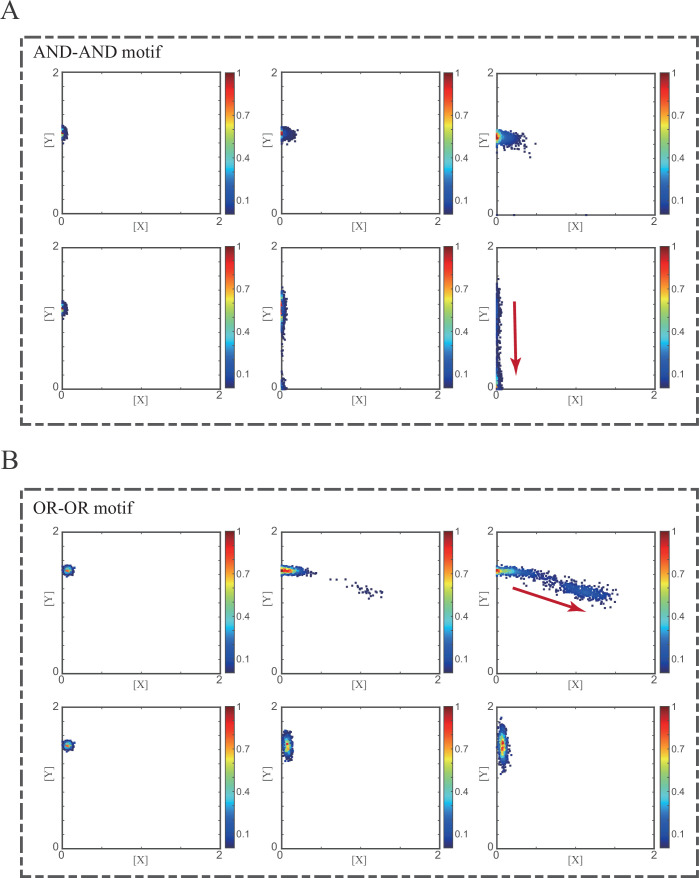
Noise as a driving force for reprogramming of LY to S in two logic motifs. (**A**) Stochastic simulation in the AND-AND motif. Initial values were identical with the attractor of LY fate in Figure 2C top panel (stable steady state, SSS in purple attractor basin). Simulation was preformed 1000 times, with each final state recorded as a dot on the plot. Top panel: Noise level of *X* (*σ_x_*) is set to 0.03, 0.09, 0.15, from left to right, and *σ_y_* is 0.03. Bottom panel: Noise level of *Y* (*σ_y_*) is set to 0.03, 0.09, 0.15, from left to right, and *σ_x_* is 0.03. Red arrow represents the direction of fate transitions of LY to S. Other than adding a white noise, parameters were identical with those in Figure 2C top panel. Color of heatmap corresponds to the density of points. (**B**) Stochastic simulation in the OR-OR motifs. Initial values were identical with attractor of LY fate in Figure 2C bottom panel (SSS in purple attractor basin). Simulation was preformed 1000 times, with each final state recorded as a dot on the plot. Top panel: Noise level of *X* (*σ_x_*) is set to 0.03, 0.09, 0.15, from left to right, and *σ_y_* is 0.03. Bottom panel: Noise level of *Y* (*σ_y_*) is set to 0.03, 0.09, 0.15, from left to right, and *σ_x_* is 0.03. Red arrow represents the direction of fate transitions of LY to S. Other than adding a white noise, parameters were identical with these in Figure 2C bottom panel.

Next, we wondered whether noise could act as a driving force for reprogramming (e.g., from LY to S). We assigned LY state as the starting cell type in simulation. Apparently, in the AND-AND motif, transition of LY to S can be realized by increasing the noise level of TF *Y* (Figure 3—figure supplement 1A). Meanwhile, in the OR-OR motif, it is the increased noise level of *X* that can drive the transition from LY to S (Figure 3—figure supplement 1B), which is also intuitive by viewing the basin geometry of the state space (Figure 2C). These observations suggested that under the noise-driven mode, experimental reprogramming strategy need to take consideration of the regulatory logic (e.g., in reprogramming of LY to S, perturb the high expression TF of LY in the AND-AND motif, while in the OR-OR motif, perturb the low expression TF).

### Section 3: Two logic motifs decide oppositely between differentiation and maintenance under the signal-driven mode

In addition to noise, cell fate decisions can also be driven by signals, e.g., GM-CSF in hematopoiesis (Mojtahedi et al., 2016), CHIR99021 in chemically induced reprogramming (Zhao, 2019). The change conducted by signals corresponds to the distortions of the cell fate landscape. To simulate the signal-driven mode, we focused on the effect of parameters in the mathematical models on the system’s dynamical properties. To simulate models feasibly and orthogonally, we added parameters *u* (*u_x_* in Equation 3, *u_y_* in Equation 4) to Equations 1; 2:

(3) $$\frac{d\left[X\right]}{dt}=\frac{r_{0}^{x}+r_{1}k_{1}{\left[X\right]}^{n_{1}}+r_{2}k_{2}{\left[Y\right]}^{n_{2}}+r_{3}k_{3}{\left[X\right]}^{n_{1}}{\left[Y\right]}^{n_{2}}}{1+k_{1}{\left[X\right]}^{n_{1}}+k_{2}{\left[Y\right]}^{n_{2}}+k_{3}{\left[X\right]}^{n_{1}}{\left[Y\right]}^{n_{2}}}-d_{1}\left[X\right]+u_{x}$$

(4) $$\frac{d\left[Y\right]}{dt}=\frac{r_{0}^{y}+r_{4}k_{4}{\left[Y\right]}^{n_{2}}+r_{5}k_{5}{\left[X\right]}^{n_{1}}+r_{6}k_{6}{\left[X\right]}^{n_{1}}{\left[Y\right]}^{n_{2}}}{1+k_{4}{\left[Y\right]}^{n_{2}}+k_{5}{\left[X\right]}^{n_{1}}+k_{6}{\left[X\right]}^{n_{1}}{\left[Y\right]}^{n_{2}}}-d_{2}\left[Y\right]+u_{y}$$

The increase of *u* represents an elevation in the basal expression level of lineage-specifying TFs, reflecting an induction signal from the extracellular environment. From an experimental standpoint, this signal can be the induction of small molecules or overexpression by gene manipulations, such as the transfection of cells with expression vectors containing specific genes.

**Figure 4.**
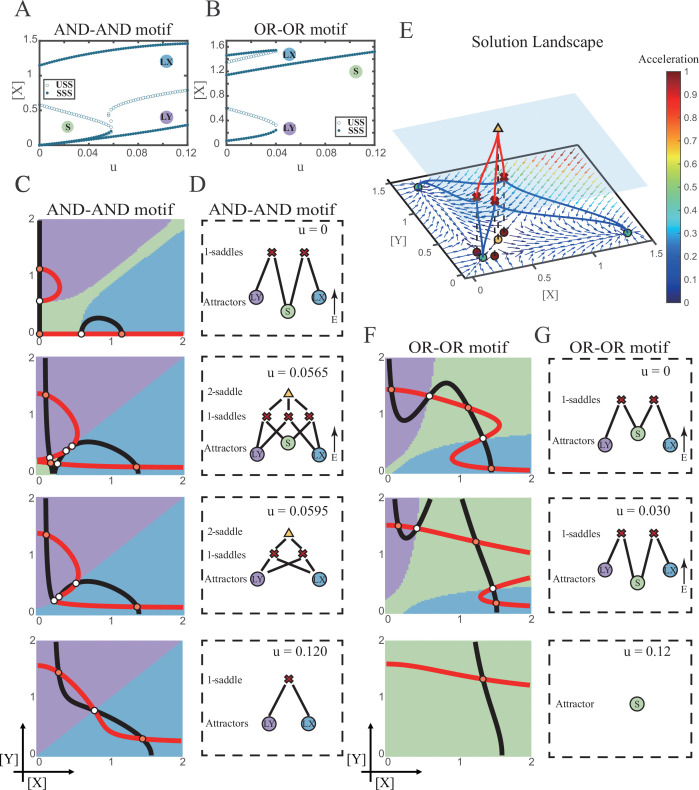
Two logic motifs decide oppositely between differentiation and maintenance under the signal-driven mode. (**A–B**) Bifurcation diagrams for the AND-AND motif (**A**) and OR-OR motif (**B**) driven by parameter *u* (*u*=*u_x_*=*u*_*y*_) in the cross-inhibition with self activation (CIS) model. Stable steady states (SSS) and unstable steady states (USS) are denoted as solid dots and hollow dots, respectively. (**C and F**) Changes in the state spaces for the AND-AND motif (**C**) and OR-OR motif (**F**) with increasing parameter *u*, from top to down. (**D and G**) Changes in the solution landscape with increasing of *u*, in company with these in (**C and F**). The crimson X-cross sign and yellow triangle denote first-order and second-order saddle nodes, respectively. Relative energy is quantified by the geometric minimum action method (Vanden-Eijnden and Heymann, 2008), see ‘Materials and methods’. (**E**) The solution landscape with parameter *u*=0.0565 for the AND-AND motif from a view of three dimensions. It describes a hierarchical structure of the steady states. From top to bottom, it represents 2-saddle (yellow triangle), 1-saddles (crimson X-cross sign), and the attractors (green dot). The layer of 1-saddles is represented by a blue translucent plane, and the bottom layer is the flow field diagram. The connections from 2-saddle to 1-saddles are represented by red lines, and the connection from 1-saddles to the attractors are represented by blue lines. In the flow field diagram, the direction and color of the arrows correspond to the direction and size of the flow at that location. The corresponding positions of 2-saddle and 1-saddles in the flow field are marked with yellow and red dots, respectively, with black dashed lines indicating the corresponding relationship.

We first explored the impact on the system when the two induction parameters are changed symmetrically (*u*=*u_x_*=*u_y_*). As the increase of *u*, the number of SSS in the AND-AND system decreases from three to two, where S attractor evaporates after a subcritical pitchfork bifurcation (Figure 4A and Figure 4—figure supplement 1A). Whereas in the OR-OR motif, after the increase of *u*, LX and LY attractors disappear with saddle-node bifurcations, respectively. Only the SSS representing stem cell fate is retained in the state space (Figure 4B). We then portrayed all the topology of the steady-state adjacency that accompanied the increase of *u,* from the perspective of the solution landscape. In the AND-AND motif, the attractor basin of LX and LY started to adjoin and occupied the vanishing S attractor basin together (Figure 4C and D). Accordingly, the stem cells cannot maintain themselves and decided to differentiate into either one of the lineages. Moreover, if the cell population possesses the same noise levels in both *X* and *Y*, then the fate decisions are unbiased (Figure 4—figure supplement 1B).

**Figure 4—figure supplement 1.**
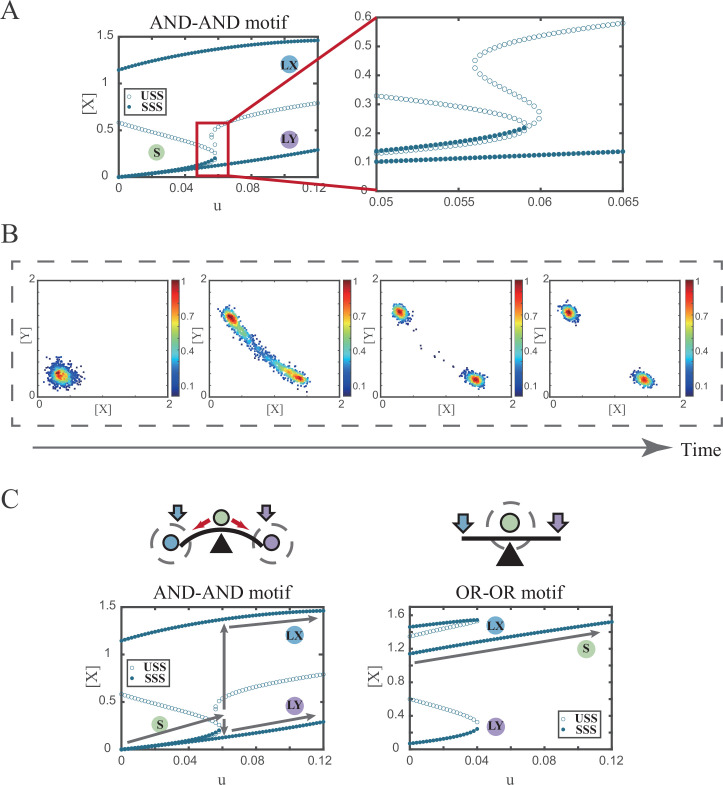
Two logic motifs are associated with opposite fate-decision choices under bidirectional induction. (**A**) Bifurcation diagrams for the AND-AND motif (Figure 4A) driven by parameter *u* (*u*=*u_x_*=*u_y_*) in the cross-inhibition with self activation (CIS) model. Stable steady states (SSS) and unstable steady states (USS) are denoted as solid dots and hollow dots, respectively. (**B**) Stochastic simulation in the AND-AND motif. Initial values were identical with attractor of S fate in Figure 2C top panel (SSS in green attractor basin). Noise level of *X* (*σ_x_*) and *Y* (*σ_y_*) are both set to 0.06. Simulation was preformed 1000 times for each pseudo-time point, with each temporal state (from left to right) recorded as a dot on the plot. Model’s parameters were identical with those in Figure 4C fourth panel. (**C**) Schematic illustration of the ‘seesaw’ model (Shu et al., 2013) in two logic motifs under bidirectional induction. Red arrows represent the direction of fate transitions. Blue and purple arrows represent induction of *X* and *Y*, respectively. Gray hollow circles indicate remained cell states. Gray arrows represent fate transitions from S in bifurcation diagrams as increasing *u*.

Notably, in the AND-AND motif, we observed a brief intermediated stage before S attractor disappears, where all three fates are directly interconnected (Figure 4C second panel and Figure 4D second panel and Figure 4E). To manifest the generality, we globally screened 6231 groups of parameter sets under the AND-AND motif, and this logic-dependent intermediated stage can be observed for 82.7% of them (see ‘Materials and methods’; Supplementary file 1), indicating little dependence on particular parameter setting (1.8% in the OR-OR motif). Unlike the indirect attractor adjacency structure mediated by S attractor (Figure 2D), the solution landscape with fully-connected structure facilitates transitions between any two pairs of fates. Furthermore, this transitory fully-connected stage is located between the fate-undetermined stage (Figure 4C top panel) and fate-determined stage (Figure 4C third panel), comparable to the initiation (or activation) stage before the lineage commitment in experimental observations (Brand and Morrissey, 2020; Zhang et al., 2018; Arinobu et al., 2007). Therefore, we suspected that the robust fully-connected stage in the AND-AND motif may correspond to a specific period in cell fate decisions.

From the standpoint of reprogramming of differentiated cells back into progenitors, in the AND-AND motif, differentiated cells are more capable of maintaining their own fates during the symmetrical increase of the induction signals on both lineages (Figure 4A and C). Whereas in the OR-OR motif, the attractor basin of LX or LY is progressively occupied by the stem cell fate as *u_x_* and *u_y_* increase together (Figure 4F and G). In this scenario, the downstream fates are eventually reversed back to the undifferentiated state (Figure 4B and F). Namely, reprogramming engaged in the OR-OR motif can be accomplished by bi-directional induction of downstream antagonistic fates. In sum, we found that under symmetrical signal induction, the behavior of stem cells is subject to core GRN’s logic motifs. In the AND-AND motif, stem cells prefer to differentiate, while under the OR-OR motif, the stem cell population inclines to maintain its undifferentiated state.

### Section 4: The trade-off between progression and accuracy of cell fate decisions under the signal-driven mode

**Figure 5.**
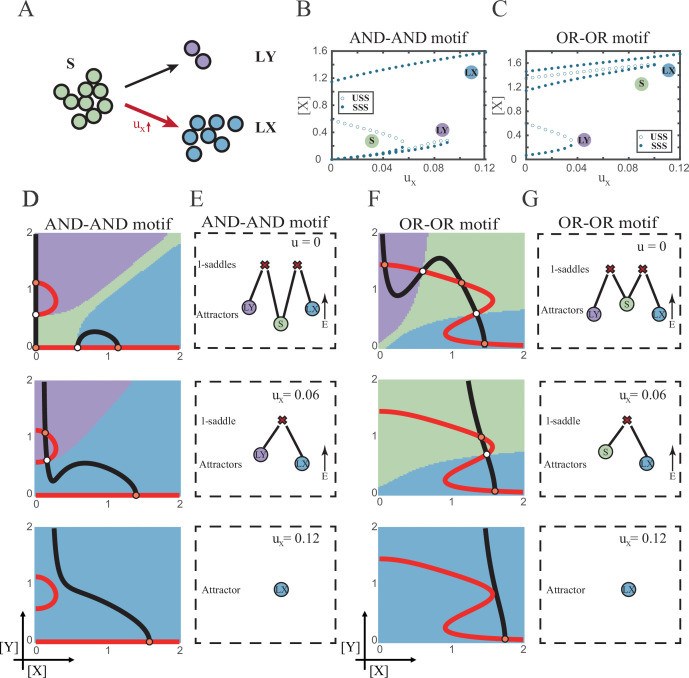
The progression-accuracy trade-off in cell fate decisions. (**A**) Schematic illustration of S-to-LX cell fate decisions with *X*-inducing signals. The red and bold arrow indicates the direction of fate decisions. (**B–C**) Bifurcation diagrams for the AND-AND motif (**B**) and OR-OR motif (**C**) driven by parameter *u_x_*. (**D and F**) Changes in the state spaces for the AND-AND motif (**D**) and OR-OR motif (**F**) with increasing values of *u_x_*, from top to down. (**E and G**) Changes in the solution landscape with increasing of *u_x_*, in company with these in (**D and F**).

According to experimental observations, the majority of fate decisions exhibit lineage preference, also known as ‘symmetry breaking of fate decisions’ (Stanoev and Koseska, 2022; Pei et al., 2020; Psaila et al., 2020; Chen et al., 2018). Take the lineage choices in hematopoiesis as an example, Some HSCs prefer myeloid over lymphoid (Pei et al., 2020; de Haan and Lazare, 2018). This fate-decision bias also further shifts along with aging and infection (Zhang et al., 2018; Reya et al., 2001). In studying this preference in fate decisions, we broke the symmetry in the signal-driven models, by solely increasing *u_x_* while keeping *u_y_*=0 (Figure 5A). First, it is apparent that the fate decision will significantly steer toward LX along with the increase of *u_x_*, regardless of the logic motifs. Ultimately the state spaces contain only LX attractor when *u_x_* is sufficiently high (Figure 5B and C and Figure 5—figure supplement 1A). However, the changes in the state space and the solution landscape follow different routes for two logic motifs. In the AND-AND motif, S attractor basin disappears at first, leaving a state space with two differentiated fates (Figure 5D and E). Then the basin of LY attractor shrinks and finally disappears (Figure 5D and E). Whereas in the OR-OR motif, LY attractor disappears first. Then S attractor, with an enlarged basin, shares the state space with LX attractor. Finally, S attractor basin abruptly disappears by a saddle-node bifurcation (Figure 5F and G).

**Figure 5—figure supplement 1.**
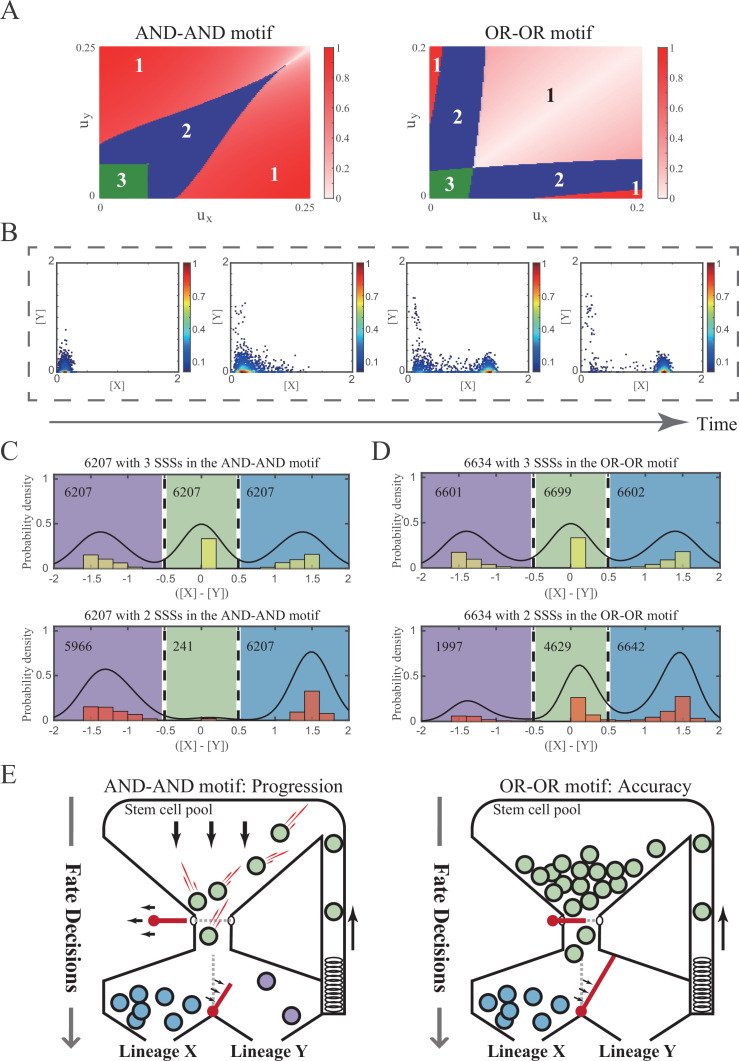
The PA (progression and accuracy) trade-off of cell fate decisions under the signal-driven mode. (**A**) Phase portraits *u_x_* vs. *u_y_* for the AND-AND and OR-OR motifs. The numbers in separating regions indicate the number of SSS. The extent of red color is quantified by minus of [X] and [Y], which indicates the expression level of balance between *X* and *Y* genes. (**B**) Stochastic simulation in the AND-AND motif. Initial values were identical with attractor of S fate in Figure 2C top panel (SSS in green attractor basin). Noise level of *X* (*σ_x_*) and *Y* (*σ_y_*) are set to 0.05, 0.15, respectively. Simulation was preformed 1000 times for each pseudo-time point, with each temporal state (from left to right) recorded as a dot on the plot. Model’s parameters were identical with those in Figure 5D middle panel. (**C–D**) Distribution of SSSs under all parameter sets in two logic motifs. We collected parameter sets with three SSSs, where two USSs are remained by increasing *u_x_* (see ‘Materials and methods’). Minus of [X] and [Y] of each SSS is quantified to represent relative cell fate LX, S, LY. Dark lines are kernel density estimation of each distribution. The numbers in separating regions indicate the number of SSS. (**E**) Schematic illustration of PA trade-off. Left panel: the entire red latch of stem cell pool is undone, which indicates all the stem cells are engaged in differentiation with fate bias (the red bar between LX and LY). Right panel: the gate for differentiating into LY is inaccessible, which indicates stem cells ‘flow’ into LX exclusively. Style was inspired by Chen et al., 2018 and Goldberg et al., 2007.

**Figure 5—figure supplement 2.**
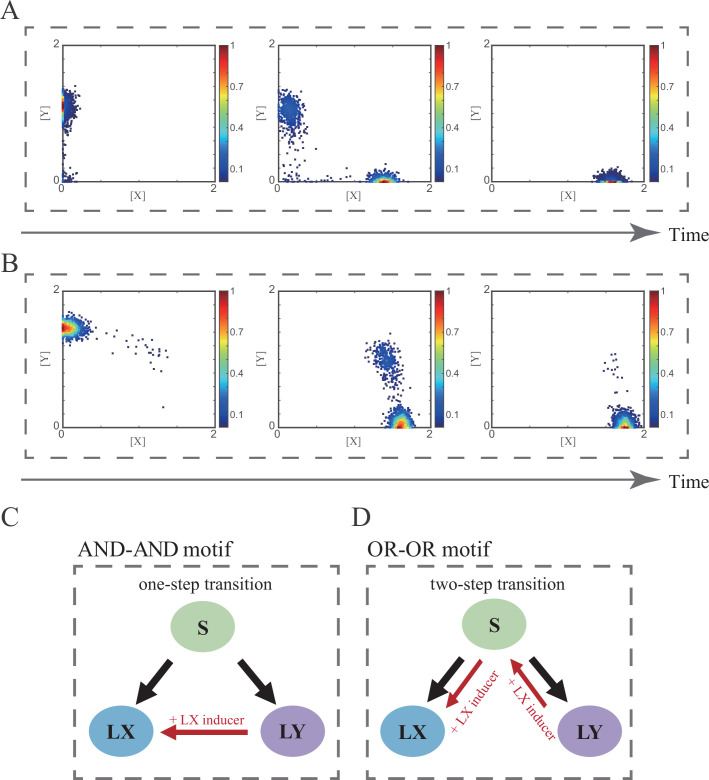
Distinct trajectories of reprogramming under the signal-driven mode. (**A–B**) Stochastic simulation in the AND-AND (**A**) and OR-OR (**B**) motifs. Initial values were identical with attractor of LY fate in Figure 2C (SSSs in purple attractor basins). Noise level of *X* (*σ_x_*) and *Y* (*σ_y_*) are both set to 0.09. Simulation was preformed 1000 times, with each final state recorded as a dot on the plot. Parameter *u_x_* switched from 0 to 0.12 (0, 0.06, 0.12, from left to right). Other model’s parameters were identical with those in Figure 2C. (**C–D**) Schematic illustration of trajectories of reprogramming in the AND-AND (**C**) and OR-OR (**D**) motifs. Dark arrows indicate in vivo differentiation from stem cell population. Red arrows indicate trajectories of trans-differentiation under the induction of *X*.

The distinct sequences of attractor basin disappearance as *u_x_* increasing can be viewed as a trade-off between progression and accuracy. In the AND-AND motif, the attractor basin of LX and LY adjoins (Figure 5D middle panel) when S attractor disappears due to the first saddle-node bifurcation (Figure 5B). Notwithstanding the bias of differentiation toward LX, the initial population still possesses the possibility of transiting into LY (Figure 5—figure supplement 1B). That is, in the AND-AND motif, as the increase of induction signal *u_x_*, the ‘gate’ for the stem cell renewal is closed first. Stem cells are immediately compelled to make fate decisions toward either LX or LY, with a bias toward LX but a nonignorable probability of entering LY. Albeit the accuracy of differentiation is, therefore, compromised, the overall progression of differentiation is ensured (i.e., all stem cells have to make the fate decisions downward. This causes the pool of stem cells to be exhausted rapidly). Whereas in the OR-OR motif, the antagonistic fate, LY, disappears first (Figure 5C). The attractor basin of S and LX are adjacent in the state space (Figure 5F). In this case, the orientation of the fate decisions is generally unambiguous since the stem cell population can only shift to LX, ensuring the accuracy of differentiation. Next, to check if the observed sequences of basin disappearance are artifacts of specific parameter choice, we randomly sampled parameter sets to check the sequence of attractor changes in their state spaces (6207 groups of the AND-AND motifs and 6634 groups of the OR-OR motifs; Supplementary file 1). We found that 96% AND-AND motifs and 70% OR-OR motifs exhibit the same sequence of attractor vanishment mentioned above (Figure 5—figure supplement 1C and D and D; see ‘Materials and methods’). These results of the global screen demonstrated that the sequence of attractor vanishment is robust to parameter settings. In sum, we proposed that logic motifs couple the trade-off between progression and accuracy as a general phenomenon in the signal-driven asymmetrical fate decisions (Figure 5—figure supplement 1E).

Next, we examined the trans-differentiation from LY into LX by increasing *u_x_*. In the AND-AND motif, with the induction of *X*, LY directly transited into LX as the stem cell state disappears before LY (Figure 5D and Figure 5—figure supplement 2A). Intriguingly, for the OR-OR motif under the same induction, LY population first returned to the S state and then flows into LX (Figure 5F and Figure 5—figure supplement 2B). Namely, different logic motifs conduct distinct trajectories in response to identical induction in reprogramming. The AND-AND motif renders a one-step transition between downstream fates (Figure 5—figure supplement 2C). While in the OR-OR motif, it is a two-step transition mediated by the stem cell state (Figure 5—figure supplement 2D). This phenomenon suggests the observation that cells may be reprogrammed to distinct cell types depending on the induction dose (Zhao, 2019) is more realizable in the OR-OR motif. Integrated with the foregoing symmetrical induction, we recapitulated that in the OR-OR motif, the bi-directional induction or a unilateral induction from a counterpart (e.g., solely induced *Y* to realize reprogramming of LX to S) confer downstream cell fates to return to the undifferentiated state (Figure 4F and Figure 5F). Whereas in the AND-AND motif, it is substantially more difficult to achieve de-differentiation. This observation may explain why some cell types are not feasible to reprogram (Shi et al., 2017).

### Section 5: The CIS network performs differently during hematopoiesis and embryogenesis

In prior sections, we systematically investigated two logic motifs under the noise- and signal-driven modes in silico. With various combinations of logic motifs and driving forces, features about fate-decision behaviors were characterized by computational models. Next, we questioned whether observations in computation can be mapped into real biological systems. And how to discern different logic motifs and driving modes is a prerequisite for answering this question.

**Figure 6.**
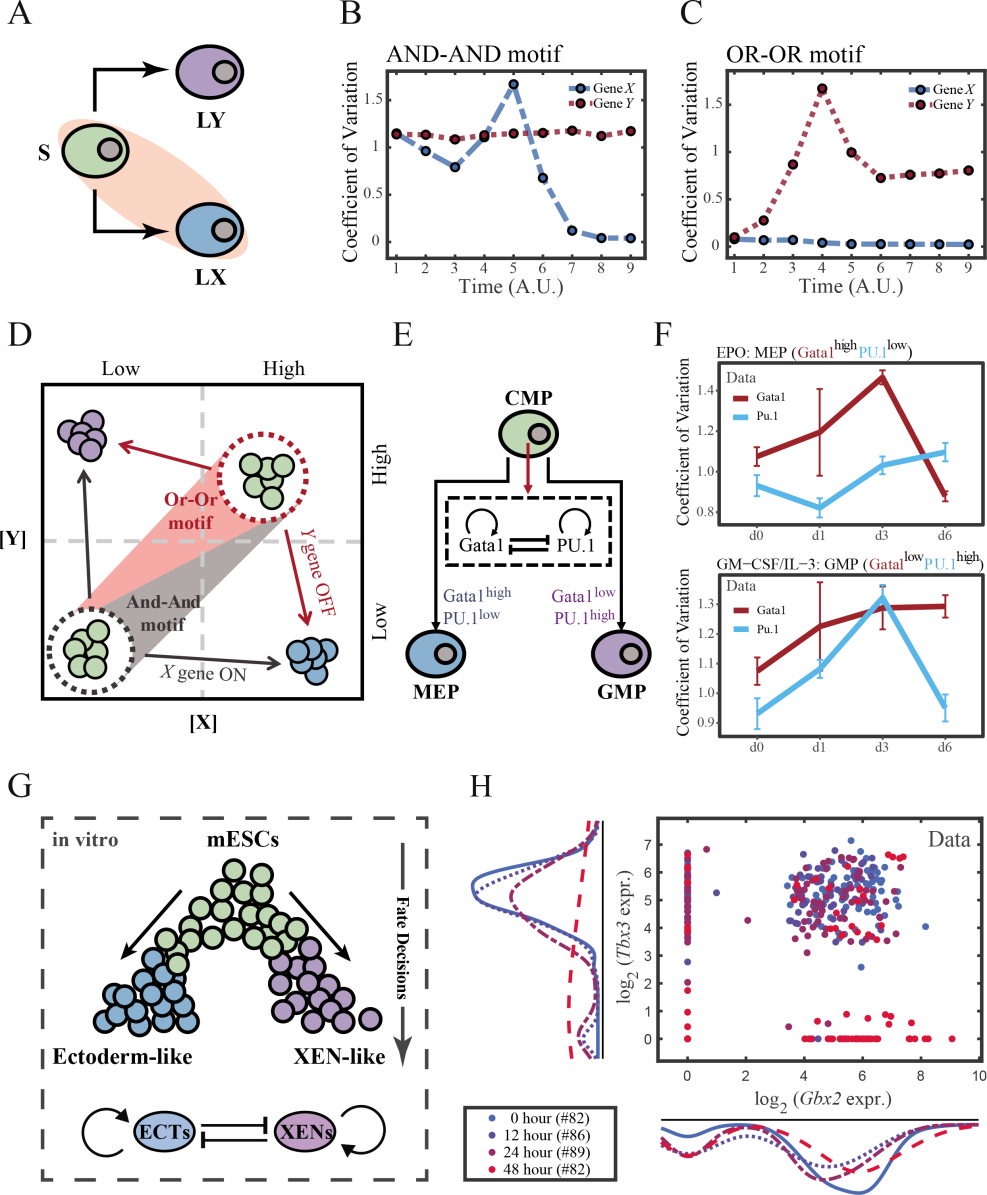
The cross-inhibition with self activation (CIS) network performs differently during hematopoiesis and embryogenesis. (**A**) Schematic illustration of S differentiating into LX. We took fate transition labeled in light pink shade as an example in the following simulation. (**B**) Time courses on the coefficient of variation in expression levels of *X* and *Y* genes in silico during differentiation towards LX (*u_x_* switches from 0 to 0.08 from time point 1–9) in the AND-AND motif. Initial values were set to the attractors of stem cell fate in Figure 2C top panel (SSS in green attractor basin). *σ_x_* and *σ_y_* are both set to 0.07. Stochastic simulation was preformed 1000 times for each pseudo-time point. Unit of time is arbitrary. (**C**) Time courses on the coefficient of variation in expression levels of *X* and *Y* genes in silico during differentiation towards LX (*u_x_* switches from 0 to 0.24 from time point 1–9) in the OR-OR motif. Initial values were set to the attractors of stem cell fate in Figure 2C bottom panel (stable steady state, SSS in green attractor basin). *σ_x_* and *σ_y_* are both set to 0.05. Stochastic simulation was preformed 1000 times for each pseudo-time point. Unit of time is arbitrary. (**D**) Schematic illustration of distinctive cell fate decision patterns under the AND-AND and OR-OR motifs in the state space. Dark and red gradients represent the extent of ‘AND-AND’ and ‘OR-OR’ in the actual regulatory network, respectively. Each axis represents expression levels of the lineage-specifying transcription factors. Blue, green, and purple circles indicate the cell fates of LX, S, and LY, respectively. (**E**) Schematic illustration of *Gata1-PU.1* circuit that dominates the primary fate decisions in hematopoiesis (CMP: Common myeloid progenitor; MEP: megakaryocyte-erythroid progenitor; GMP: Granulocyte-monocyte progenitor). (**F**) Measured coefficient of variation of expression levels of *Gata1* and *PU.1* changing over time during differentiation from CMPs to MEPs and GMPs. Expression levels were quantified via single-cell RT-qPCR (Mojtahedi et al., 2016). Error bars on points represent standard deviation (SD). For details of data processing, see ‘Materials and methods’. (**G**) Schematic illustration of the differentiation from mESCs in an induction system (Semrau et al., 2017). (**H**) Measured expression levels of *Gbx2* and *Tbx3* among cells in embryogenesis quantified via single-cell SMART-seq2 (Semrau et al., 2017). For details of data processing, see ‘Materials and methods’.

To end this, we first evaluated the performance of different models, specifically in simulating the process of stem cells differentiating towards LX (Figure 6A). Under four models with different combinations of driving modes and logic motifs (Figure 6—figure supplement 1A and B), we assessed the expression level and expression variance (defined as the coefficient of variation) of TFs *X* and *Y* among the cell population over time in stochastic simulation. We observed that, under the same logic motifs, different driving modes change in the patterns of expression variance rather than expression levels (Figure 6B and C and Figure 6—figure supplement 1C and D). Overall, under the noise-driven differentiation from S to LX, the variance of expression exhibits a continuous and monotonic trend (Figure 6—figure supplement 1D) for both logic motifs. For different logic motifs, in the AND-AND motif, the expression variance of *X* (highly expressed in LX) declines (Figure 6—figure supplement 1D top panel). Whereas in the OR-OR motif, it is the expression variance of *Y* (low expressed in LX) displays a rising trend (Figure 6—figure supplement 1D bottom panel). Nevertheless, under the signal-driven mode, the expression variance increases and then decreases, exhibiting a non-monotonic transition due to signal-induced bifurcation. During S to LX differentiation, comparable to the noise-driven mode, it is the expression variance of TF *X* in the AND-AND motif and TF *Y* in the OR-OR motif display a nonmonotonic pattern.

**Figure 6—figure supplement 1.**
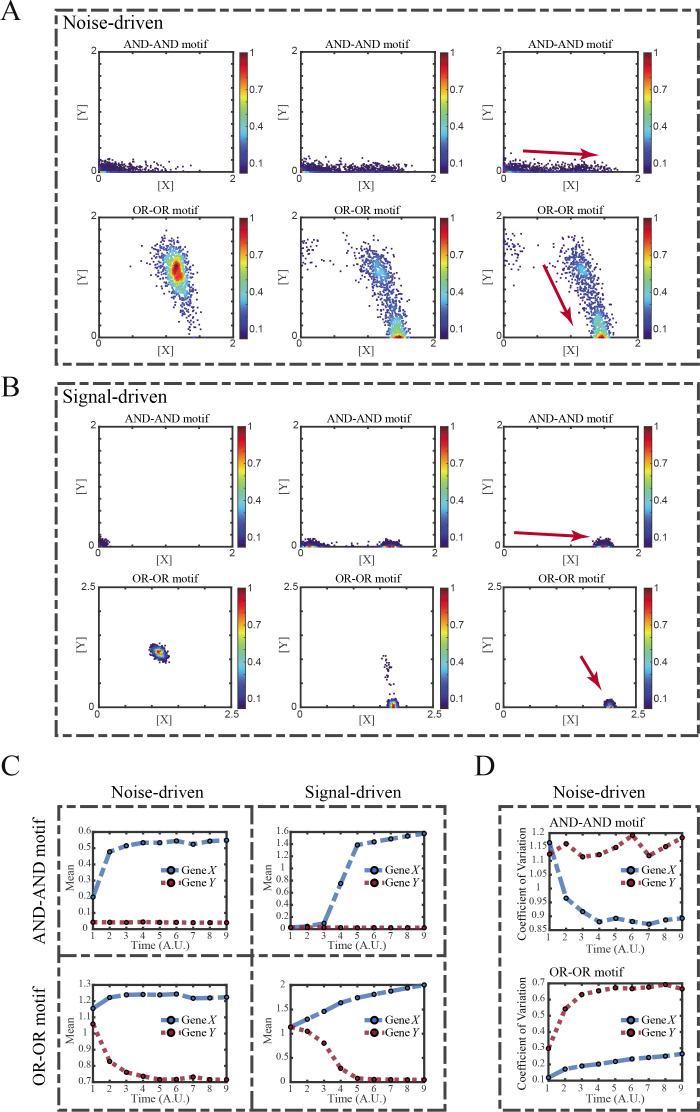
Simulation of fate transitions of S to LX in silico in two logic motifs under two driving forces. (**A**) Simulation in two logic motifs under the noise-driven mode. Initial values were identical with attractor of S fate in Figure 2C (Stable steady states, SSSs in green attractor basins). Simulation was preformed 1000 times for each pseudo-time point, with each temporal state (from left to right) recorded as a dot on the plot. Top panel: Noise level of *X* (*σ_x_*) is set to 0.21, and *σ_y_* is 0.09. Bottom panel: Noise level of *Y* (*σ_y_*) is set to 0.21, and *σ_x_* is 0.09. Red arrow represents the direction of fate transitions of S to LX. Other than adding a white noise, parameters were identical with those in Figure 2C. (**B**) Simulation in two logic motifs under the signal-driven mode. Initial values were identical with attractor of S fate in Figure 2C (SSSs in green attractor basins). Top panel: Noise level of *X* (*σ_x_*) and *Y* (*σ_y_*) are both set to 0.06. Simulation was preformed 1000 times, with each final state recorded as a dot on the plot. Parameter *u_x_* switched from 0 to 0.09 (0, 0.045, 0.09, from left to right). Bottom panel: Noise level of *X* (*σ_x_*) and *Y* (*σ_y_*) are both set to 0.05. Simulation was preformed 1000 times, with each final state recorded as a dot on the plot. Parameter *u_x_* switched from 0 to 0.24 (0, 0.12, 0.24, from left to right). Red arrow represents the direction of fate transitions of S to LX. Other model’s parameters were identical with those in Figure 2C. (**C**) Time courses on the mean in expression levels of *X* and *Y* genes in silico during differentiation towards LX. Initial values were set to the attractors of S fate in Figure 2C (SSSs in green attractor basins). Simulation of the noise-driven mode in two logic motifs was identical with that in Figure 6—figure supplement 1A. Simulation of the signal-driven mode in two logic motifs was identical with that in Figure 6—figure supplement 1B (Parameter *u_x_* switched from 0 to 0.12 from time point 1–9 in the AND-AND motif). (**D**) Time courses on the coefficient of variation in expression levels of *X* and *Y* genes in silico during differentiation towards LX under the noise-driven mode. Initial values were set to the attractors of S fate in Figure 2C (SSSs in green attractor basins). Simulation of the noise-driven mode in two logic motifs was identical with that in Figure 6—figure supplement 1A.

**Figure 6—figure supplement 2.**
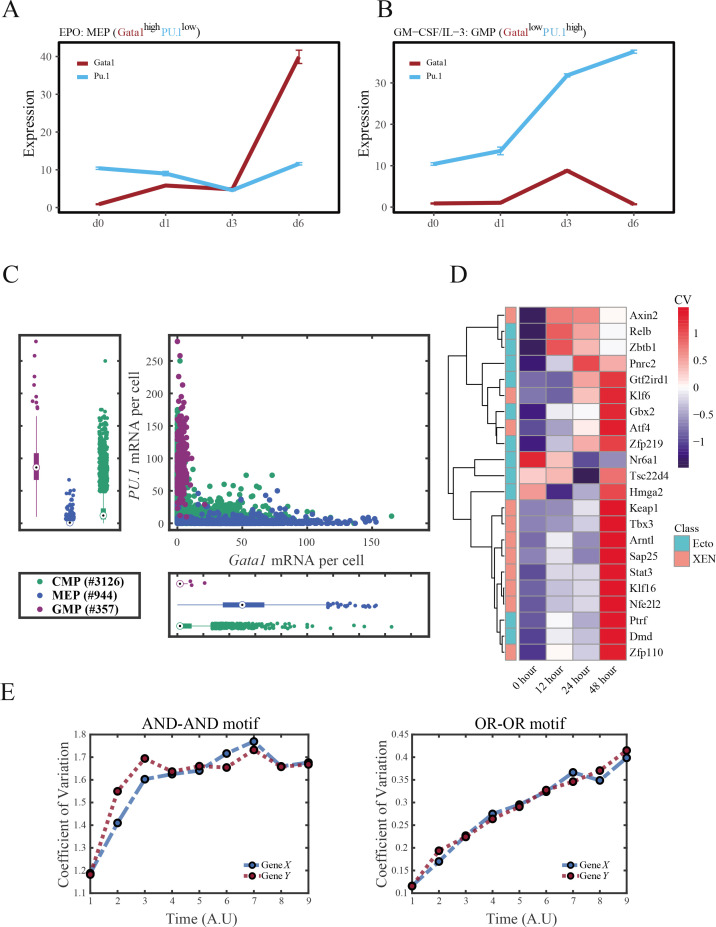
Gene regulatory networks (GRNs) perform differently in differentiations during hematopoiesis and embryogenesis. (**A–B**) Measured mean of expression levels of *Gata1* and *PU.1* changing over time in the processes of differentiation from Common myeloid progenitors (CMP) to megakaryocyte-erythroid progenitors (MEP) and Granulocyte-monocyte progenitors (GMP). Expression levels were quantified via single-cell RT-qPCR (Mojtahedi et al., 2016). For details of data processing, see ‘Materials and methods. (**C**) Expression level of *Gata1* and *PU.1* among CMPs, MEPs, and GMPs quantified via single-molecule RNA fluorescent in situ hybridization (Wheat et al., 2020). (**D**) Heatmap of coefficient of variation of expression levels of 22 genes among cells in embryogenesis quantified via single-cell SMART-seq2 (Semrau et al., 2017). For details of data processing, see ‘Materials and methods’. (**E**) Time courses on the coefficient of variation in expression levels of *X* and *Y* genes in silico during differentiation under the noise-driven mode. Initial values were set to the attractors of S fate in Figure 2C (Stable steady states, SSSs in green attractor basins). Top panel: Noise level of *X* (*σ_x_*) and *Y* (*σ_y_*) are both set to 0.14. Bottom panel: Noise level of *X* (*σ_x_*) and *Y* (*σ_y_*) are both set to 0.1. Stochastic simulation was preformed 1000 times for each pseudo-time point.

Such disparities between logic motifs originate from the location of S attractor (Figure 6D and Figure 2C). Although the target cell types are the same (LX), the AND-AND motif requests the expression of the TF *X* to be turned on, while the OR-OR motif requests the TF *Y* to be turned off. These key fate-transition genes, namely TF *X* in the AND-AND motif and TF *Y* in the OR-OR motif, both exhibit a sharp increase of variation in response to saddle-node bifurcation driven by *u_x_* induction (first saddle node in Figure 5B; second saddle node in Figure 5C). Overall, these computational results suggest that we may be able to distinguish the two driving modes according to the expression variance over time series, then logic motifs can be correspondingly assigned by the expression level of the genes in the target cell types. For instance, if the expression variance of the *X* gene exhibits a nonmonotonic pattern and *X* is highly expressed in target cell types, then this cell fate decision can be assigned as the signal-driven fate decision in an AND-AND-like motif.

To support our findings with real-world correspondence, we first focused on the differentiation of CMPs in hematopoiesis (Figure 6E). It is acknowledged that the transcriptional regulation of *Gata1-PU.1* circuit dominates this cell fate decisions, which conforms to the CIS topology (Figure 6E). Mojtahedi et al., 2016 stimulated murine multipotent hematopoietic precursor cell line EML with erythropoietin (EPO) or granulocyte-macrophage colony-stimulating factor/interleukin 3 (GM-CSF/IL-3) to examine the commitment into an erythroid or a myeloid fate, respectively. Based their curated dataset, we found that the expression level of *Gata1* (highly expressed in MEPs) gradually increased during EPO induction (Figure 6—figure supplement 2A), while the expression variance exhibits a nonmonotonic trend (Figure 6F top panel). Symmetrically, during GM-CSF/IL-3 induced differentiation toward GMPs, the expression level of *PU.1* (highly expressed in GMPs) gradually increased (Figure 6—figure supplement 2B), while the expression variance also presents a nonmonotonic pattern (Figure 6F bottom panel). The trends shown in the dataset resemble the signal-driven mode with the AND-AND motif. In addition, we quantified the expression of *Gata1* and *PU.1* via the single molecule FISH dataset (Figure 6—figure supplement 2C; Wheat et al., 2020). They are at low levels in CMPs, corresponding to the expression patterns in the AND-AND motifs (Figure 2E and Figure 6D). Together, we suggested that the *Gata1-PU.1* circuit performs in an AND-AND-like manner, and this differentiation system (Mojtahedi et al., 2016) is under the signal-driven mode.

Another paradigmatic model of fate decision is the differentiation of embryonic stem cells (ESC). Semrau et al., 2017 found that under the retinoic acid (RA) exposure system in vitro, mouse embryonic stem cells (mESC) differentiated into two lineages: extraembryonic endoderm (XEN)-like and ectoderm-like. The investigators recapitulated that two clusters of TFs with the CIS topology determined this lineage specification (Figure 6G). We observed that the expression variance in most of these fate-decision TFs (16/22 73%) are gradually increasing during time, and 14% (3/22) of them exhibit nonmonotonic behavior (Figure 6—figure supplement 2D), suggesting the process is more likely driven by noise (Figure 6—figure supplement 2E). Furthermore, we focused on potential key regulators: *Gbx2* and *Tbx3*, the two likely targets of RA that are crucial for this fate decision (Semrau et al., 2017). The expression variances over time of these two TFs are consistently increasing (Figure 6—figure supplement 2D). In addition, their initial expressions are at a high level, in agreement with that of the OR-OR motif (Figure 6D and H). In short, we proposed that the mESCs differentiation system under RA exposure performs in an OR-OR-like manner, and its differentiation is under the noise-driven mode in this experimental setting.

### Section 6: The chemical-induced reprogramming of human erythroblasts (EB) to induced megakaryocytes (iMK) is the signal-driven fate decisions with an OR-OR-like motif

**Figure 7.**
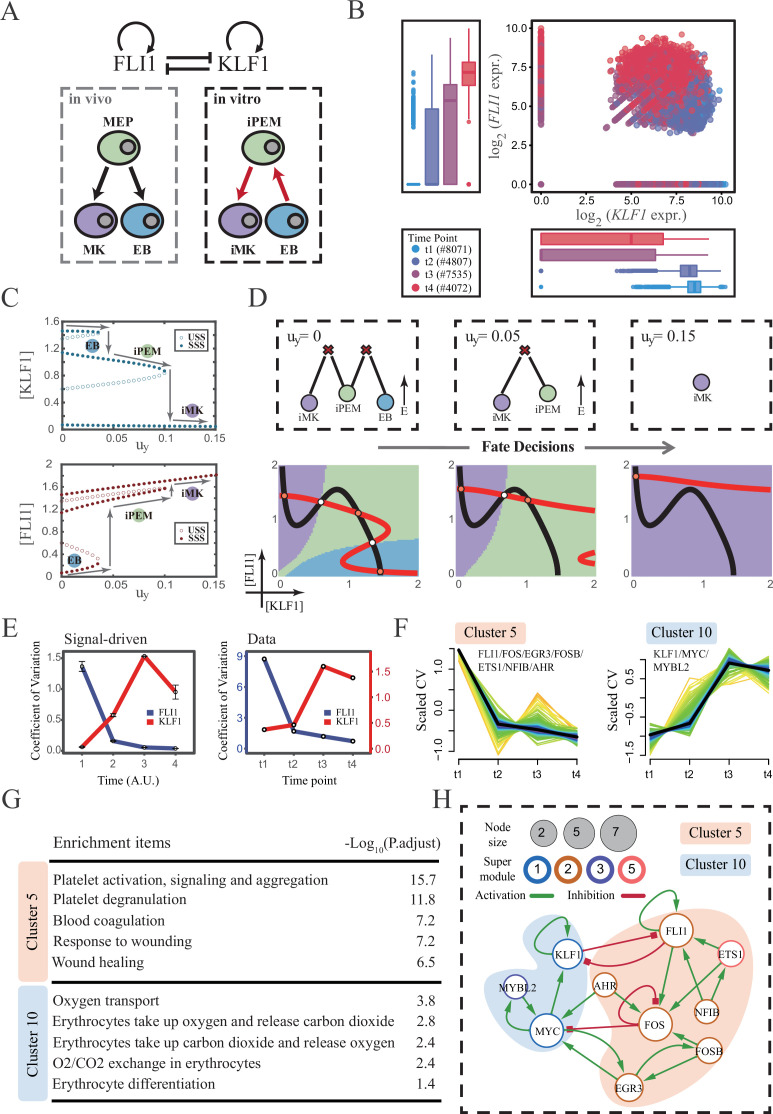
The chemical-induced reprogramming of human erythroblast (EB) to induced megakaryocyte (iMK) is the signal-driven fate decisions with an OR-OR-like motif. (**A**) Schematic illustration of the differentiation from megakaryocyte-erythroid progenitors (MEPs) in vivo and in vitro.Red arrows represent the route of reprogramming (Qin et al., 2022) (**B**) Measured expression levels of *KLF1* and *FLI1* in reprogramming quantified via single-cell 10 X. For details of data processing, see ‘Materials and methods’. (**C**) Bifurcation diagrams for the OR-OR motif driven by parameter *u_y_* in the CIS model. (**D**) Fate transition representing reprogramming of EB to iMK in silico. Top panel: changes in the solution landscape with increasing of parameter *u_y_*, from left to right; Bottom panel: changes in the state spaces for the OR-OR motif with increasing values of *u_y_*, in company with these in top panel. Unit of concentration is arbitrary. (**E**) Left panel: coefficient of variation of expression levels of *KLF1* and *FLI1* changes in silico over time under given parameter (*u_y_*=0.11) in the OR-OR motif. Noise level of *KLF1* (*σ_x_*) and *FLI1* (σ_y_) are set to 0.087. Initial values were identical with LX attractor in Figure 2C bottom panel (stable steady state, SSS in blue attractor basin). Stochastic simulation was preformed 1000 times per round for each time point. We totally preformed three round simulations. Error bars on points represent standard deviation (SD); Right panel: measured coefficient of variation of expression levels of *KLF1 and FLI1* changing over time in the processes from EBs to iMKs. Unit of time is arbitrary. (**F**) Identification of distinct temporal patterns of expression variance by fuzzy c-means clustering. The x-axis represents four time points, while the y-axis represents scaled CV (coefficient of variation) in each time point. Dark trend lines in the middle indicate the average of scaled CV over genes in cluster. (**G**) Enriched major Gene Ontology terms for clusters 5 and 10. (**H**) Regulatory network of transcription factors (TFs) in cluster 5 and 10. Circle size indicates the sum of in-degree and out-degree. Node colors indicate different Supermodules (adapted from Qin et al., 2022). Green and red edges indicate activation and inhibition, respectively. The light blue and light pink shades denote genes in clusters 5 and 10, respectively.

The foregoing cell fate decisions initiate from pluripotent cells in mice (mESC, CMP) corresponding to a typical ‘downhill’ in Waddington’s metaphor. In 2006, Yamanaka et al. accomplished the reprogramming from mouse fibroblasts into iPSC state via the noted ‘OSKM’ factors, representing ‘uphill’ in Waddington’s metaphor (Shi et al., 2017; Hamazaki et al., 2017; Abdallah and Del Vecchio, 2019). Likewise, trans-differentiations from one lineage to another have been realized by overexpression or chemical inductions (Xu et al., 2015), whether they correspond to direct ‘trespassing’ of the ridge or an ‘up-and-down’ through the peak in Waddington landscape are still elusive. We then applied our models to reprogramming systems, with a primary focus on hematopoiesis. Qin et al., 2022 recently achieved the direct chemical reprogramming of EBs to iMKs using a four-small-molecule cocktail (Figure 7A). Investigators presented that EBs underwent an induced bipotent precursor for erythrocytes and MKs (iPEM) to finally desired iMKs. It is acknowledged that the *FLI1-KLF1* circuit with the CIS topology dominates this fate-decision process (Orkin and Zon, 2008; Palii et al., 2019). To deduce the logic motif of the *FLI1-KLF1* circuit, we quantified the expression patterns of *FLI1* and *KLF1* based on published single-cell RNA-seq data (Qin et al., 2022). We can observe the fate transition from the EB population (*FLI1*^low^, *KLF1*^high^) to the iMK population (*FLI1*^high^, *KLF1*^low^) (Figure 7B). According to their expression level, the cell populations can be primarily classified into three clusters. In addition, both *FLI1* and *KLF1* are highly expressed in the intermediate cell population suspected to be the progenitors of iMKs and EBs (Qin et al., 2022). Namely, the pattern of expression level is concordant with the OR-OR motif in our framework (Figure 6D).

**Figure 7—figure supplement 1.**
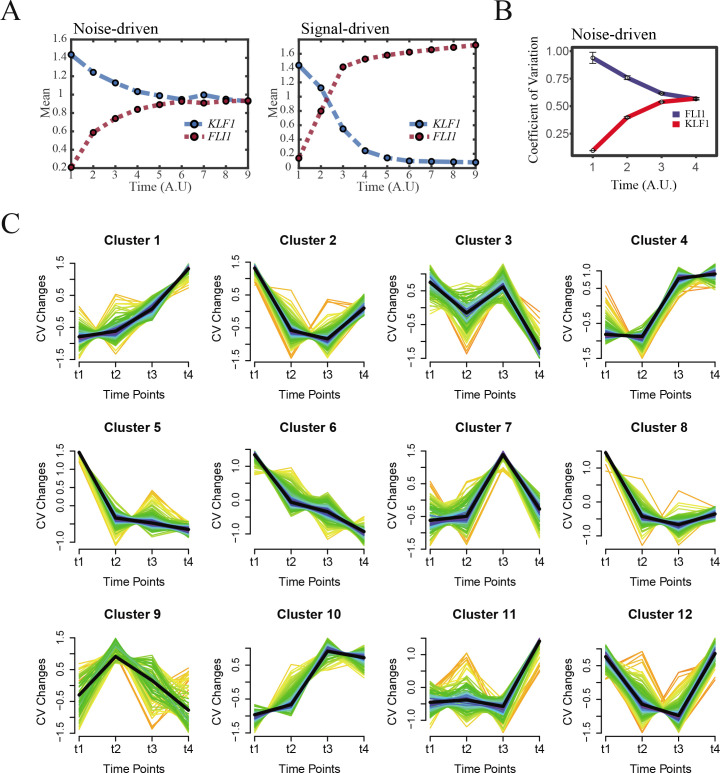
The chemical-induced reprogramming of human erythroblast (EB) to induced megakaryocyte (iMK) is the signal-driven fate decisions. (**A**) Time courses on the mean in expression levels of *KLF1* and *FLI1* genes in silico during reprogramming of EBs to iMKs under the noise-driven (left) and signal-driven (right) modes. Initial values were set to the attractors of LX fate in Figure 2C bottom panel (SSS in blue attractor basin). Noise level of *KLF1* (*σ_x_*) and *FLI1* (*σ_y_*) are both set to 0.18 under the noise-driven mode. Stochastic simulation was preformed 1000 times for each pseudo-time point. Other than adding a white noise, parameters were identical with those in Figure 2C bottom panel. Simulation of the signal-driven mode was identical with that in Figure 7E left panel. (**B**) Coefficient of variation of expression levels of *KLF1* and *FLI1* changes in silico along by pseudo-time under the OR-OR motif. Simulation was identical with that in Figure 7—figure supplement 1 left panel. (**C**) Identification of distinct temporal patterns of expression variance by fuzzy c-means clustering. The x-axis represents four time points, while the y-axis represents scaled CV (coefficient of variation) in each time point. Dark trend lines in the middle indicate average of scaled CV over genes in cluster.

Next, to investigate the driving force of this reprogramming system, we simulated the fate transition from EB (corresponding to LX, blue) to iMK (LY, purple) under both driving modes. Under the noise-driven mode, we assumed that the reprogramming system facilitated the noise levels of both TFs. For simplicity, the starting cell population (EBs) was assigned symmetrical high noise levels. While under the signal-driven mode, we assumed that the four-small-molecule cocktail upregulated the expression of *FLI1* (highly expressed in iMKs). Then, we simulated the transition from EBs to iMKs by lifting the basal expression level of *FLI1*, corresponding to parameter *u_y_* in model (Figure 7C). The bifurcation diagrams indicate that the signal-driven fate transition is mediated by the iPEM state (Figure 7C). In particular, overexpression of *FLI1* renders sequential saddle-node bifurcations. Thus, EBs are converted to iPEMs before steering toward the terminal iMK state (Figure 7C and D), which is consistent with the experiment’s findings.

Furthermore, we next assessed the dynamic behaviors in models of this system under different driven modes with the OR-OR logic. As discussed before, there is no discernible difference in the expression levels between the two driving modes. Overall, the common tendency is an up-regulation in *KLF1* and a down-regulation in *FLI1* (Figure 7—figure supplement 1A). We then quantified the expression variances during this fate transition by the model. Under the noise-driven mode, expression variances of *FLI1* and *KLF1* would gradually decrease and increase, respectively, until stabilizing (Figure 7—figure supplement 1B). Under the signal-driven mode, the expression variance of *FLI1* would first decline and then remain nearly constant, while *KLF1* would exhibit a nonmonotonic pattern (Figure 7E left panel). From the view of modeling, the nonmonotonic pattern presented by *KLF1* originates from the rapid shut-off during the transition from iPEM state to iMK state (second saddle node in Figure 7C).

Accordingly, we next quantified the expression variances in the real dataset over time. Impressively, the pattern emerging from the data accommodates the hypothesis of the signal-driven mode (Figure 7E). Altogether, we proposed that this reprogramming system (Qin et al., 2022) is the signal-driven process underlying the OR-OR-like motif. Moreover, the high expression level of *FLI1* induced by small molecules is the key driving force of the fate transition, as suggested by the properties of the OR-OR motif. We underlined that it is a classical two-step fate-decision process mediated by the upstream progenitor state, which is in agreement with the phenomenon articulated by Qin et al., 2022.

We then searched for genes with similar patterns of expression variance to those of *KLF1* and *FLI1*, with a hypothesis that genes possessing comparable expression variance patterns, especially TFs, may synergistically perform fate-decision related functions. Thus, we applied the fuzzy c-means algorithm (Kumar and Futschik, 2007) to cluster genes based on their expression variances, rather than expression levels. In total, we observed 12 distinct clusters of temporal patterns (Figure 7—figure supplement 1C; Supplementary file 2). We focused primarily on clusters 5 and 10, where *KLF1* and *FLI1* are found respectively (Figure 7F). To testify our hypothesis, we conducted an enrichment analysis using the gene set in clusters 5 and 10. Functions associated with specific cell types are significantly enriched (Figure 7G). In particular, cluster 5 with *FLI1* is largely related to platelet-related functions, whereas cluster 10 with *KLF1* is largely related to the energy metabolism of blood cells. Furthermore, we filtered out 15 TFs in clusters 5 and 10 (11 in cluster 5; 4 in cluster 10). Next, by harnessing the TF interaction database (see ‘Materials and methods’; Supplementary file 3), we collected 21 regulons associated with 10 TFs to construct the TF regulatory Network (Figure 7H). Intriguingly, in the original article, genes were classified into six ‘Supermodules’ according to the patterns of expression levels. Genes in cluster 5 are located in Supermodules 1 and 3, representing a decreasing tendency for expression level. Meanwhile, genes in cluster 10 are distributed in Supermodules 2 and 5, and their expression levels raise from low to high (Figure 6D from Qin et al., 2022). Of note, most of the TFs filtered by expression variance patterns appear in the GRN constructed in the original article (8/10, 80%; Figure 6G from Qin et al., 2022). As a result, we underscored that *EGR3* and *ETS1*, which did not appear in the GRN of the original article, have been suggested to play important roles in the trans-differentiation. In addition, we observed that *MYC* and *FOS* possess the largest connectivity in our 10-node GRN, suggesting that these two TFs as hubs are essential regulators of this reprogramming system.

Together, in mapping our framework to the real reprogramming system, we assigned the cell fate decisions of EBs to iMKs to the signal-driven mode incorporated the OR-OR-like motif, by comparing the expression and expression variance patterns measured from the real dataset with pseudo-data produced by our models in a ‘top-down’ fashion. According to the model, the reprogramming is primarily driven by induced up-regulation of FLI1. Additionally, from the view of expression variance, we recapitulated a concise 10-node GRN and identified some TF nodes not previously recognized as major regulators, like *EGR3* and *ETS1*.

## Discussion

Comprehending the driving forces behind cell fate decisions is crucial for both fundamental scientific research and biomedical engineering. Recent advances in data collection and statistical methods have greatly enhanced our understanding of the mechanisms that regulate cell fate. However, despite the widespread use of the Waddington landscape as a metaphor in experiments, there has been little examination into whether the fate decision observed in a particular experiment corresponds to a stochastic shift from one attractor to another on the landscape (i.e., noise-driven) or an overall distortion of the landscape (i.e., signal-driven). The application of appropriate computational models in systems biology can aid in uncovering the underlying mechanisms (Cahan et al., 2021; del Sol and Jung, 2021). One of the most representative work is that Huang et al., 2007 modeled the bifurcation in hematopoiesis to reveal the lineage commitment quantitatively. Compared to simply modularizing activation or inhibition effect by employing Hill function in previous work, our models reconsidered the multiple regulations from the level of TF-CRE binding.

Our computational investigations have emphasized the importance of the combinatorial logic in connecting gene expression patterns with the driving forces underlying cell fate decisions. Utilizing a representative network topology known as the CIS, our analysis demonstrated how both driving forces and regulatory logic jointly shape expression patterns during fate transitions. In turn, mean and variance in gene expression patterns can reveal logic motifs and driving forces. Our analytical framework promotes the interpretability of fate decisions and can be employed to speculate on the driving factors of fate decisions using a ‘top-down’ approach, thereby providing a reference for investigating the causality of fate decisions and experimental validation.

The roles of noise as a possible driver of fate transitions are intriguing. By our models, the relationship among noise configuration, logic motifs, and fate-decision bias has been unveiled, and we noticed the opposite fate bias for the AND-AND and OR-OR motifs. Conversely, on the demand of the same bias, the progenitors with different logic motifs tend to employ a different profile of noise level (Figure 3E and F). Therefore, we suggested that under the noise-driven mode, the logic motif works like a ‘broker’ to shape the fate preferences. Based on the assumption that the preference of fate decisions is the result of evolution and adaptation, we posited that if an organism has a functional demand for a specific bias, the noise profile will be iterated via logic motifs. In turn, changes in noise levels will be mediated by logic motifs to shape the differentiation bias. One intuitive example is the significant shift in differentiation preferences of HSCs over aging (López-Otín et al., 2013; Beerman and Rossi, 2015; Dorshkind et al., 2020). It has been reported that aging HSCs show different levels of DNA methylation and epigenetic histone modifications from young HSCs (de Haan and Lazare, 2018; Kovtonyuk et al., 2016; Pang et al., 2017). How changes in epigenetic level shape the noise profile of the cell population and further affect the shift of fate-decision preference is a fascinating question. We underscored that the dissection of logic motifs underlying associated GRNs is a prerequisite for answering that question.

With the ever-expanded of single-cell sequencing data, characterizing genes by their mean expressions does not make the most of high-throughput datasets. As an intrinsic characteristic in central dogma, expression variance has been utilized to locate the ‘critical transitions’ in complex networks (Wang and Chen, 2020; Scheffer et al., 2012), e.g., identify the critical transitions in diseases like lymphoma (Liu et al., 2012). Instead of differentially expressed genes, Rosales-Alvarez et al., 2022 harnessed differentially noisy genes to characterize the functional heterogeneity in HSC aging. Our work presents that the patterns of expression variance can also be used to indicate driving forces and key regulators during the fate-decision processes. Nevertheless, compared to traditional gene expression, the interpretation of expression variance patterns is generally not intuitively accessible (Grün et al., 2014). Additionally, extra a priori knowledge is needed to filter out the cluster of interest. To this end, our framework enables researchers to locate functional clusters via mathematic model based on appropriate hypothesis. Notably, if the genes that constituting the CIS network are not specified, we can conversely leverage the patterns of temporal expression variance to nominate key regulators in a model-guided manner. Collectively, our framework provides a mechanistic explanation for expression variance patterns and qualitatively characterize key expression variance patterns to locate core regulators of fate decisions without reliance on a priori knowledge.

Comparing to tuning noise, altering signals is a more accessible approach for experimentally manipulating cell fates. When the basal expressions of two lineage-specifying genes grow symmetrically, we have shown that opposite trends of fate transitions occur under two logic motifs: In the AND-AND motif, it promotes differentiation, whereas the OR-OR motif stabilizes stem cell fates. This is reminiscent of the ‘seesaw’ model where maintenance of stemness can be achieved by overexpression of antagonistic lineage-specifying genes (Shu et al., 2013). Our model suggests that restoring stemness by inducing two antagonistic lineage-specifying genes is more likely under the OR-OR-like motif (Figure 4—figure supplement 1C). This is in concert with our analysis that mESC differentiation system performs in an OR-OR-like manner. In addition, Mojtahedi et al., 2016 found that, under simultaneous induction of two antagonistic fates (EPO and GM-CSF/IL-3), although differentiation was delayed, it eventually occurred. This observation is consistent with models in the AND-AND motif, and we suggested that the core regulatory circuits in hematopoiesis performs in an AND-AND-like manner. More experimental validations would be needed to validate this hypothesis that ‘the seesaw model prefers the OR-OR motif.’ Conversely, insight from the ‘seesaw’ model also provides a candidate approach to further testify logic motifs underlying GRNs in experiments. For instance, stem cells with an AND-AND-like circuit are expected to display differentiation rather not maintenance in response to bidirectional induction.

In quantifying the signal-driven landscape changes, the solution landscape enables intuitive interpretation even in high-dimension GRNs (Yin et al., 2020; Sáez et al., 2022; Corson and Siggia, 2012). In this work, we used both the state space and the solution landscape, in order to relate them for further investigations involving more than two TFs. Interestingly, from the perspective of the solution landscape, we found a robust fully-connected stage in the AND-AND motif. We envisioned that this period corresponds to the priming stage of differentiation. Notably, this fully-connected stage was not found in the OR-OR motif, suggesting that the necessity for priming during differentiation may be subject to the logic motifs of core GRNs.

Actual cell fate decisions are seldom purely unbiased. Under the asymmetrical signal-driven mode, we summarized the progression-accuracy trade-off in cell fate decisions: If a large number of cells are ensured to differentiate, then concessions have to be made in the accuracy of differentiation, and vice versa (Figure 5—figure supplement 1E). An intuitive example is the large-scale apoptosis occurs daily in hematopoiesis. Hence, maintaining homeostasis in vivo inevitably requests cells to respond rapidly to differentiation. A recent study reported that in response to systemic inflammation by polymicrobial sepsis, pool of CMPs is rapidly depleted to accelerate the production of downstream cell fates (Fanti et al., 2023). This is concordant with our result that *Gata1-PU.1* circuit in hematopoiesis performs in an AND-AND-like manner. On the other hand, in embryonic development like *C. elegans*, the accuracy of cell fate decisions is considerably emphasized. How this nature of differentiation has been adapted in evolution is an interesting question. Our work highlights that this property is associated with the logic motifs of GRNs, suggesting the emphasis on progression or accuracy may be embedded in the logic motifs of core GRNs.

We classified three examples of cell fate decisions based on patterns of expression and expression variance. In hematopoiesis, we took fate choice between erythroid and myeloid as a paradigm, and assigned it an AND-AND-like motif under the signal-driven mode. In embryogenesis, we suggested the fate decision in RA exposure system is an OR-OR-like motif under the noise-driven mode. In reprogramming, the chemical-induced trans-differentiation is the signal-driven fate decisions incorporated an OR-OR-like motif. For simplicity and intuitiveness, we devised our model with two symmetrical combinations of regulatory logic (AND-AND/OR-OR). Albeit there are merely four types of cell fate decisions in consideration, our framework enables to be generalized and expanded to accommodate multi-node GRNs and complex logic combinations. Plenty of studies zoomed in one particular fate-decision events. However, from the standpoint of systems biology, we underlined that classification of fate decisions is a vital step for further investigation, as is the case for the typing of cells and tumors. Theoretically, appropriate classification of fate-decision systems enables the enrichment of common properties. So, accumulated knowledge can be inherited to new fate-decision cases. Taking reprogramming as an example, Zhao, 2019 recapitulated five kinds of trajectories in chemical-induced reprogramming. We suggested that the reprogramming trajectory is coupled with the logic motifs. On one hand, it is possible to answer why a certain reprogramming system exhibit a particular trajectory. On the other hand, it is possible to postulate achievable reprogramming according to the logic motifs of core GRNs (e.g., the AND-AND motif is more likely to enable direct conversion; model 4 mentioned in Zhao, 2019). Recently, synthetic biology has realized the insertion of the CIS network in mammalian cells (Zhu et al., 2022). One of the prerequisites for recapitulating the complex dynamics of fate transitions in synthetic biology is systematical understanding of the role of GRNs and driving forces in differentiation. And the logic motifs are the essential and indispensable elements in GRNs. Our work also provides a blueprint for designing logic motifs with particular functions. We are also interested in validating the conclusions drawn from our models in a synthetic biology system.

### Limitation of this study

Although our framework enables the investigation of more logic motifs, we chose two classical and symmetrical logic combinations for our analysis. Future work should involve more logic gates like XOR and explore asymmetrical logic motifs like AND-OR. The gene expression datasets analyzed here are only available for a limited number of time points. Though they meet the need for discerning trends, it is evident that the application to the datasets with more time points will yield clearer and less ambiguous changing trends to support the conclusions of this paper more generally. Notwithstanding the fact that the CIS network is prevalent in fate-decision programs, there are other topologies of networks that serve important roles in the cell-state transitions, like feed-forward loop, etc. The framework should further incorporate diverse network motifs in the future. In addition, for simplicity and intuition, we here considered signals as uncoupled and additive effects in ODE models, due to feasible mapping in real biological systems, such as ectopic overexpression.

## Materials and methods

### Derivation of the CIS network

In GRN, each TFs is represented by a node and the edge between nodes represents the regulatory relationship. In our work, we considered a GRN comprised of 2 TFs (i.e., X, Y), formulated with 2 ODEs describing the change of each TF. Define ([X], [Y]) to be the concentration of TF X and Y, the ODE model is described as follows:

(5) $$\left\{ \begin{array}{l} \frac{d[X]}{dt}=H_{X}([X],\;[Y])-d_{X}[X]\\ \frac{d[Y]}{dt}=H_{Y}([X],\;[Y])-d_{Y}[X] \end{array}\right.$$

where H_X_([X], [Y]) is the production rate of TF *X* that combines the effects from both activators and suppressors of the X and *d_X_* is the decay rate from the *X* and *Y*, which integrate degradation and dilution.

The exact form of H_X_([X], [Y]), H_Y_([X], [Y]) are derived based on the simple GRN with nodes *X* and *Y* via a set of molecular interactions between these TFs themselves, genes that encode for them, and the mRNAs (Abdallah and Del Vecchio, 2019).

TFs in a GRN act as multimers to implement regulatory interaction. In our model, we treated TFs *X* and *Y* as acting in their homo-multimer forms, X_n1_ and Y_n2_, respectively (i.e., *n_1_* TF X monomers reversibly form an activated homo-multimerized form X_n1_ and *n_2_* monomers of TF Y to reversibly form activated homo-multimer Y_n2_). Of note, *n_1_* and *n_2_* here are able to be further generalized beyond the number of binding elements (Nam et al., 2022; Santillán, 2008). The multimerization biochemical reaction of TFs X and Y can be represented as follows,

(6) $$\underset{n_{1}\;monomers}{\underbrace{X+X+\cdot \cdot \cdot +X}}\overset{q_{x}}{\underset{p_{x}}{⇌}}X_{n_{1}},\;\underset{n_{2}\;monomers}{\underbrace{Y+Y+\cdot \cdot \cdot +Y}}\overset{q_{y}}{\underset{p_{y}}{⇌}}Y_{n_{2}}$$

There are many diverse mechanisms of regulation (Lambert et al., 2018; Balsalobre and Drouin, 2022). For simplicity, our model considered transcriptional regulation as a major mechanism since it is feasibly characterized in experiments and can well represent the interaction between genes in Cross-Inhibition with Self-activation (CIS) network. To activate downstream transcription, TFs bind with their Cis-Regulatory Elements (CREs). We denoted D_X_ for the no-bound CREs of TF X and D_Y_ for the TF Y in an independent manner. Here, we posited different TFs bind to exclusive, non-overlapping CREs to regulate target genes. Hence, there are totally eight binding patterns described as follows,

(7) $$\begin{array}{cc} D_{X}+X_{n_{1}}\overset{P_{1}}{\underset{q_{1}}{⇌}}D_{XX}, & D_{X}+Y_{n_{2}}\overset{P_{2}}{\underset{q_{2}}{⇌}}D_{XY},\\ D_{Y}+Y_{n_{2}}\overset{P_{4}}{\underset{q_{4}}{⇌}}D_{YY}, & D_{Y}+X_{n_{1}}\overset{P_{3}}{\underset{q_{3}}{⇌}}D_{YX},\\ D_{XX}+Y_{n_{2}}\overset{P_{5}}{\underset{q_{5}}{⇌}}D_{XXY}, & D_{XY}+X_{n_{1}}\overset{P_{6}}{\underset{q_{6}}{⇌}}D_{XXY},\\ D_{YX}+Y_{n_{2}}\overset{P_{7}}{\underset{q_{7}}{⇌}}D_{YYX}, & D_{YY}+X_{n_{1}}\overset{P_{8}}{\underset{q_{8}}{⇌}}D_{YYX}. \end{array}$$

Next, we modeled the biochemical reactions of transcription and translation. Transcription is an elaborate process involving many steps, including initiation, elongation, and termination of an mRNA transcript. Likewise, translation includes peptide formation and elongation, followed by protein folding before function. However, to evaluate the behavior of GRN concisely, we treated transcription and translation as single-step reactions and then use lumped rates to encompass the time it takes for all steps in the elaborate machinery to complete (Abdallah and Del Vecchio, 2019). Hence, the transcription process can be represented as follows,

(8) $$\begin{array}{ccc} & D_{XX}\overset{\alpha_{x}^{1}}{\to }m_{X}+D_{XX}, & D_{YY}\overset{\alpha_{y}^{1}}{\to }m_{Y}+D_{YY},\\ D_{X}\overset{\alpha_{x}}{\to }m_{X}+D_{X}, & D_{XY}\overset{\alpha_{x}^{2}}{\to }m_{X}+D_{XY}, & D_{YX}\overset{\alpha_{y}^{2}}{\to }m_{Y}+D_{YX},\\ D_{Y}\overset{\alpha_{y}}{\to }m_{Y}+D_{Y}, & D_{XXY}\overset{\alpha_{x}^{3}}{\to }m_{X}+D_{XXY}, & D_{YYX}\overset{\alpha_{y}^{3}}{\to }m_{Y}+D_{YYX}. \end{array}$$

Here, we distinguished the basal rate of transcription of a gene without activation or repression (termed as constitutive transcription). The basal transcription rate is shown in the left panel in Equation 8.

Though we considered eight configurations, these ultimately led to two mRNA transcripts, *m_X_* and *m_Y_*. Translation can also be represented by a one-step process from these transcripts to their protein products. The one-step translation biochemical reactions are described as follows,

(9) $$\begin{array}{cc} m_{X}\overset{\kappa_{x}}{\to }m_{X}+X, & m_{Y}\overset{\kappa_{y}}{\to }m_{Y}+Y. \end{array}$$

Next, we considered the decay of these proteins as well as their respective mRNA species. In general, these proteins and mRNA species will undergo decay to some extent which is a combination of both degradation and dilution.

(10) $$\begin{array}{ccc} X\overset{\delta_{x}}{\to }\emptyset , & m_{X}\overset{\eta_{x}}{\to }\emptyset , & X\overset{\beta }{\to }\emptyset ,\\ Y\overset{\delta_{y}}{\to }\emptyset , & m_{Y}\overset{\eta_{y}}{\to }\emptyset , & Y\overset{\beta }{\to }\emptyset , \end{array}$$

here, the left and middle panels represent the degradation of TFs X and Y as well as mRNA transcripts *m_X_* and *m_Y_*. The right panel represents the dilution of TFs X and Y due to cell division. In general, the degradation rate γ can be determined using the following relations,

(11) $$\begin{array}{ccc} \delta =\frac{ln2}{t_{\frac{1}{2}}}, & \eta =\frac{ln2}{t_{\frac{1}{2}}}, & \beta \approx \frac{ln2}{t_{doubling}}, \end{array}$$

where *t_1/2_* represents the half-lives of each protein or mRNA transcript, *t_doubling_* represents the cell division rate. For protein products X and Y, we used the notation d to illustrate the total rate of decay rather than two separate parameters in our model, i.e., *d_1_* = *δ_x_ + β*, *d_2_* = *δ_y_ + β*, respectively. In general, *t_1/2_* and *t_doubling_* are robust and stationary, thus we treated these paramaters as constant in our model, i.e., *d_i_* = Const, for *i* = 1, 2.

We have described the reactions that comprise the endogenous components of our GRN. In our work, fate decisions are classified into two modes, i.e., driven by the noise of intracellular gene expression or driven by extracellular signals. Under the noise-driven mode, a Gaussian white noise is added to the concentration of TFs to illustrate the random intracellular noise. Whereas if driven by signals, the expression of a gene will be affected by extracellular molecular cocktails, physical stimulation, etc. Therefore, we added the production rate of the TF’s mRNA from the ectopic DNA to generalize our model.

(12) $$\begin{array}{c} \emptyset \overset{u_{x}}{\to }m_{X},\emptyset \overset{u_{y}}{\to }m_{Y}, \end{array}$$

here *u_x_*, *u_y_* represent the additional mRNA species *m_X_* and *m_Y_* via ectopic overexpression at rates *u_x_* and *u_y_*, respectively. The biochemical reactions take place on a faster timescale. This enables us to derive the change in concentration of species from a biochemical reaction based on the law of mass action. For example, for multimerization reaction of X in Equation 6, once equilibrium, we can get

(13) $$\underset{n_{1}\;monomers}{\underbrace{X+X+\cdot \cdot \cdot +X}}\overset{q_{x}}{\underset{p_{x}}{⇌}}X_{n_{1}}\Rightarrow p_{x}\underset{n_{1}\;terms}{\underbrace{\left[X][X]\cdot \cdot \cdot [X\right]}}=p_{x}[X{]}^{n_{1}}=q_{x}[X_{n_{1}}]\Rightarrow K_{x}≜\frac{p_{x}}{q_{x}}=\frac{\left[X_{n_{1}}\right]}{[X{]}^{n_{1}}}.$$

Also, for TF X bind with its CRE *D_X_*, we can get

(14) $$D_{X}+X_{n_{1}}\overset{p_{1}}{\underset{q_{1}}{⇌}}D_{XX}\Rightarrow p_{1}\left[D_{X}\right]\left[X_{n_{1}}\right]=q_{1}\left[D_{XX}\right]\Rightarrow K_{1}≜\frac{p_{1}}{q_{1}}=\frac{\left[D_{XX}\right]}{\left[D_{X}\right]\left[X_{n_{1}}\right]},$$

here, we define $K_{1}≜\frac{p_{1}}{q_{1}}$ as a constant, i.e., chemical equilibrium constant. Furthermore, we define $K_{1}≜\frac{p_{1}}{q_{1}},i=x,y,1,2,\cdots ,8$ for biochemical reactions in Equation 6 and Equation 7, respectively.

Then, we constructed an ODE model for the GRN. For a given species *S*, the principle to get a single equation is as follows:

(15) $$Change\in concentration\;of\;S=\frac{d\left[S\right]}{dt}=\left[\overset{˙}{S}\right]=\sum \left\{all\;biochemical\;recation\;rates\;involving\;S\right\}$$

To explain how this principle assists us to build the ODE model, we took species *m_X_* as a running example. Summarizing all of these biochemical reaction rates involving *m_X_* we can get the change in concentration [*m_X_*],

(16) $$\left[\overset{˙}{m_{X}}\right]=\alpha_{x}\left[D_{X}\right]+\alpha_{x}^{1}\left[D_{XX}\right]+\alpha_{x}^{2}\left[D_{XY}\right]+\alpha_{x}^{3}\left[D_{XXY}\right]-\eta_{x}\left[m_{X}\right]+u_{x}.$$

Doing so for our biochemical reaction network yielded the equations of our ODE model,

(17) $$\left\{ \begin{array}{l} \{\dot{\left[X\right]}=\kappa_{x}[m_{X}]-d_{1}[X]\;\dot{\left[Y\right]}=\kappa_{y}[m_{Y}]-d_{2}[Y]\;\dot{\left[m_{X}\right]}=\alpha_{x}[D_{X}]+\alpha_{x}^{1}[D_{XX}]+\alpha_{x}^{2}[D_{XY}]+\alpha_{x}^{3}[D_{XXY}]-\eta_{x}[m_{X}]+u_{x}\;\dot{\left[m_{Y}\right]}=\alpha_{y}[D_{Y}]+\alpha_{x}^{1}[D_{YY}]+\alpha_{x}^{2}[D_{YX}]+\alpha_{x}^{3}[D_{YYX}]-\eta_{y}[m_{Y}]+u_{y}\;\dot{\left[D_{X}\right]}=-p_{1}[X_{n_{1}}][D_{X}]+q_{1}[D_{XX}]-p_{2}[Y_{n_{2}}][D_{X}]+q_{2}[D_{XY}]\;\dot{\left[D_{Y}\right]}=-p_{4}[Y_{n_{2}}][D_{Y}]+q_{4}[D_{YY}]-p_{3}[X_{n_{1}}][D_{Y}]+q_{3}[D_{YX}]\;\dot{\left[D_{XX}\right]}=p_{1}[D_{X}][X_{n_{1}}]-q_{1}[D_{XX}]-p_{5}[D_{XX}][Y_{n_{2}}]+q_{5}[D_{XXY}]\;\dot{\left[D_{XY}\right]}=p_{2}[D_{X}][Y_{n_{2}}]-q_{2}[D_{XY}]-p_{6}[D_{XY}][X_{n_{1}}]+q_{6}[D_{XXY}]\;\dot{\left[D_{YX}\right]}=p_{3}[D_{Y}][X_{n_{1}}]-q_{3}[D_{YX}]+p_{7}[D_{YX}][Y_{n_{2}}]-q_{7}[D_{YYX}]\;\dot{\left[D_{YY}\right]}=p_{3}[D_{Y}][Y_{n_{2}}]-q_{4}[D_{YY}]+p_{8}[D_{YY}][X_{n_{1}}]-q_{8}[D_{YYX}]\;\dot{\left[D_{XXY}\right]}=p_{5}[D_{XX}][Y_{n_{2}}]-q_{5}[D_{XXY}]+p_{6}[D_{XY}][X_{n_{1}}]-q_{6}[D_{XXY}]\;\dot{\left[D_{YYX}\right]}=p_{7}[D_{YX}][Y_{n_{2}}]-q_{7}[D_{YYX}]+p_{8}[D_{YY}][X_{n_{1}}]-q_{8}[D_{YYX}]\;\dot{\left[X_{n_{1}}\right]}=p_{x}[X{]}^{n_{1}}-q_{x}[X_{n_{1}}]-p_{1}[D_{X}][X_{n_{1}}]+q_{1}[D_{XX}]-p_{3}[D_{Y}][X_{n_{1}}]+\;q_{3}[D_{YX}]-p_{6}[D_{XY}][X_{n_{1}}]+q_{6}[D_{XXY}]-p_{8}[D_{YY}][X_{n_{1}}]+q_{8}[D_{YYX}]\;\dot{\left[Y_{n_{2}}\right]}=p_{y}[Y{]}^{n_{2}}-q_{y}[Y_{n_{2}}]-p_{2}[D_{X}][Y_{n_{2}}]+q_{2}[D_{XY}]-p_{4}[D_{Y}][Y_{n_{2}}]+\;q_{4}[D_{YY}]-p_{5}[D_{XX}][Y_{n_{2}}]+q_{5}[D_{XXY}]-p_{7}[D_{YX}][Y_{n_{2}}]+q_{7}[D_{YYX}] \end{array}\right.$$

The 14-dimension ODE model of the GRN contains overwhelming parameters that make it impractical to analyze for cell fate. To reduce the dimensionality of the model, we assumed the multimerization, DNA binding/unbinding and mRNA dynamic occurs sufficiently faster than protein production and decay, the temporal derivatives of the respective species can be set to 0, indicating that the species concentration reaches its quasi-steady state (Abdallah and Del Vecchio, 2019). Thus, we can get

(18) $$\left\{ \begin{array}{l} \left[\overset{˙}{X}\right]=\kappa_{x}\left[m_{X}\right]-d_{1}\left[X\right]\\ \left[\overset{˙}{Y}\right]=\kappa_{y}\left[m_{Y}\right]-d_{2}\left[Y\right]\\ \left[\overset{˙}{m_{X}}\right]=0=\alpha_{x}\left[D_{X}\right]+\alpha_{x}^{1}\left[D_{XX}\right]+\alpha_{x}^{2}\left[D_{XY}\right]+\alpha_{x}^{3}\left[D_{XXY}\right]-\eta_{x}\left[m_{X}\right]+u_{x}\\ \left[\overset{˙}{m_{Y}}\right]=0=\alpha_{y}\left[D_{Y}\right]+\alpha_{y}^{1}\left[D_{YY}\right]+\alpha_{y}^{2}\left[D_{YX}\right]+\alpha_{y}^{3}\left[D_{YYX}\right]-\eta_{y}\left[m_{Y}\right]+u_{y}. \end{array}\right.$$

To better demonstrate the deviation of the model, we take TF X as a running example. Since GRN is symmetric, we can deviate TF Y’s equation in the same way. Once the biochemical reaction reaches its equilibrium, using the law of mass action we can get

(19) $$\begin{array}{c} {}[X_{n_{1}}]=K_{x}[X{]}^{n_{1}}\;\left[D_{XX}\right]=K_{1}\left[D_{X}\right]\left[X_{n_{1}}\right]=K_{1}\left[D_{X}\right]\left[X_{n_{1}}\right]=K_{x}K_{1}\left[D_{X}\right][X{]}^{n_{1}}\;\left[D_{XY}\right]=K_{1}\left[D_{X}\right]\left[Y_{n_{2}}\right]=K_{1}\left[D_{X}\right]\left[Y_{n_{2}}\right]=K_{y}K_{2}\left[D_{X}\right][Y{]}^{n_{2}}\;\left[D_{XXY}\right]=K_{5}\left[D_{XX}\right]\left[Y_{n_{2}}\right]=K_{5}\left[D_{XX}\right]\left[Y_{n_{2}}\right]=K_{x}K_{y}K_{1}K_{5}\left[D_{X}\right][X{]}^{n_{1}}[Y{]}^{n_{2}} \end{array}$$

Noticing that $\left[\dot{m_{X}}\right]$ = 0 in Equation 18, the species [*m_X_*] can be represented by CRE terms,

(20) $$\begin{array}{ll} \left[m_{X}\right] & =\frac{1}{\eta_{x}}\left(\alpha_{x}\left[D_{X}\right]+\alpha_{x}^{1}\left[D_{XX}\right]+\alpha_{x}^{2}\left[D_{XY}\right]+\alpha_{x}^{3}\left[D_{XXY}\right]\right)+\frac{u_{x}}{\eta_{x}}\\ & =\frac{1}{\eta_{x}}\left(\alpha_{x}\left[D_{X}\right]+\alpha_{x}^{1}K_{1}\left[D_{X}\right]\left[X_{n_{1}}\right]+\alpha_{x}^{2}K_{2}\left[D_{X}\right]\left[Y_{n_{2}}\right]+\alpha_{x}^{3}K_{1}K_{5}\left[D_{X}\right]\left[X_{n_{1}}\right]\left[Y_{n_{2}}\right]\right)+\frac{u_{x}}{\eta_{x}}\\ & =\frac{1}{\eta_{x}}\left(\left[D_{X}\right]+\alpha_{x}^{1}K_{x}K_{1}\left[D_{X}\right]{\left[X\right]}^{n_{1}}+\alpha_{x}^{2}K_{y}K_{2}\left[D_{X}\right]{\left[Y\right]}^{n_{2}}+\alpha_{x}^{3}K_{x}K_{y}K_{1}K_{5}\left[D_{X}\right]{\left[X\right]}^{n_{1}}{\left[Y\right]}^{n_{2}}\right)+\frac{u_{x}}{\eta_{x}} \end{array}$$

In our well-stirred system, for TF X’s CREs, the conservation law holds, i.e., the total CRE concentration of X, as a constant, equals the sum of CRE concentration that is bound by X, by Y, and by X and Y. By employing so and combining with Equation 19 we can get a representation of [*D_X_*],

(21) $$\frac{\left[D_{TX}\right]=\left[D_{X}\right]+\left[D_{XY}\right]+\left[D_{XX}\right]+\left[D_{XXY}\right]\Rightarrow \left[D_{X}\right]=\left[D_{TX}\right]}{\left(1+K_{x}K_{1}[X{]}^{n_{1}}+K_{y}K_{2}[Y{]}^{n_{2}}+K_{x}K_{y}K_{1}K_{5}[X{]}^{n_{1}}[Y{]}^{n_{2}}\right)}.$$

Substitute [*D_X_*] in Equation 20 with Equation 21, we can get

(22) $$\begin{array}{ll} \left[m_{X}\right] & =\frac{1}{\eta_{X}}[D_{X}](\alpha_{x}+\alpha_{x}^{1}K_{x}K_{1}[X{]}^{n_{1}}+\alpha_{x}^{2}K_{y}K_{2}[Y{]}^{n_{2}}+\alpha_{x}^{3}K_{x}K_{y}K_{1}K_{5}[X{]}^{n_{1}}[Y{]}^{n_{2}})+\frac{u_{x}}{\eta_{x}}\\ & =\frac{D_{TX}}{\eta_{x}}\frac{\left(\alpha_{x}+\alpha_{x}^{1}K_{x}K_{1}[X{]}^{n_{1}}+\alpha_{x}^{2}K_{y}K_{2}[Y{]}^{n_{2}}+\alpha_{x}^{3}K_{x}K_{y}K_{1}K_{5}[X{]}^{n_{1}}[Y{]}^{n_{2}}\right)}{\left(1+K_{x}K_{1}[X{]}^{n_{1}}+K_{y}K_{2}[Y{]}^{n_{2}}+K_{x}K_{y}K_{1}K_{5}[X{]}^{n_{1}}[Y{]}^{n_{2}}\right)}+\frac{u_{x}}{\eta_{x}}. \end{array}$$

Then finally we reach to the point that the deviation of [X]. In Equation 18 we substituted [*m_X_*] with Equation 22, and got

(23) $$\begin{array}{ll} \dot{\left[X\right]} & =\kappa_{x}[m_{X}]-d_{1}[X]\\ & =\frac{D_{TX}\kappa_{x}}{\eta_{x}}\frac{\left(\alpha_{x}+\alpha_{x}^{1}K_{x}K_{1}[X{]}^{n_{1}}+\alpha_{x}^{2}K_{y}K_{2}[Y{]}^{n_{2}}+\alpha_{x}^{3}K_{x}K_{y}K_{1}K_{5}[X{]}^{n_{1}}[Y{]}^{n_{2}}\right)}{\left(1+K_{x}K_{1}[X{]}^{n_{1}}+K_{y}K_{2}[Y{]}^{n_{2}}+K_{x}K_{y}K_{1}K_{5}[X{]}^{n_{1}}[Y{]}^{n_{2}}\right)}+\frac{\kappa_{x}u_{x}}{\eta_{x}}-d_{1}[X] \end{array}$$

here, we define $\gamma_{0}^{x}=\alpha_{x}\frac{D_{TX}\kappa_{x}}{\eta_{x}},\gamma_{1}=\alpha_{x}^{1}\frac{D_{TX}\kappa_{x}}{\eta_{x}},\gamma_{2}=\alpha_{x}^{2}\frac{D_{TX}\kappa_{x}}{\eta_{x}},\gamma_{3}=\alpha_{x}^{3}\frac{D_{TX}\kappa_{x}}{\eta_{x}}$ as relative protein generation rates, $k_{1}=K_{1}K_{x},k_{2}=K_{2}K_{y},k_{3}=K_{x}K_{y}K_{1}K_{5}$ as the weight of each TF binding patterns, as ectopic DNA (to simplify the notation, we may drop the tilde of ${\tilde{u}}_{i},i=x,y$), then we can get

(24) $$\left[\dot{X}\right]=\frac{r_{0}^{x}+r_{1}k_{1}[X{]}^{n_{1}}+r_{2}k_{2}[Y{]}^{n_{2}}+r_{3}k_{3}[X{]}^{n_{1}}[Y{]}^{n_{2}}}{1+k_{1}[X{]}^{n_{1}}+k_{2}[Y{]}^{n_{2}}+k_{3}[X{]}^{n_{1}}[Y{]}^{n_{2}}}-d_{1}[X]+u_{x},$$

Similarly, for TF Y, we can get

(25) $$\begin{array}{ll} \dot{\left[Y\right]} & =\frac{D_{TY}\kappa_{y}}{\eta_{y}}\frac{\left(\alpha_{y}+\alpha_{y}^{1}K_{y}K_{3}[Y{]}^{n_{2}}+\alpha_{y}^{2}K_{y}K_{4}[X{]}^{n_{1}}+\alpha_{y}^{3}K_{x}K_{y}K_{3}K_{7}[X{]}^{n_{1}}[Y{]}^{n_{2}}\right)}{\left(1+K_{y}K_{3}[Y{]}^{n_{2}}+K_{y}K_{4}[X{]}^{n_{1}}+K_{x}K_{y}K_{3}K_{7}[X{]}^{n_{1}}[Y{]}^{n_{2}}\right)}+\frac{\kappa_{y}u_{y}}{\eta_{y}}-d_{2}[Y]\\ & =\frac{r_{0}^{y}+r_{4}k_{4}[Y{]}^{n_{2}}+r_{5}k_{5}[X{]}^{n_{1}}+r_{6}k_{6}[X{]}^{n_{1}}[Y{]}^{n_{2}}}{1+k_{4}[Y{]}^{n_{2}}+k_{5}[X{]}^{n_{1}}+k_{6}[X{]}^{n_{1}}[Y{]}^{n_{2}}}-d_{2}[Y]+u_{y}. \end{array}$$

Ultimately, by combining Equation 24 and Equation 25, we reduced our 14-dimension model to a 2-dimension dynamic system,

(26) $$\left\{ \begin{array}{l} \frac{d[X]}{dt}=\frac{r_{0}^{x}+r_{1}k_{1}[X{]}^{n_{1}}+r_{2}k_{2}[Y{]}^{n_{2}}+r_{3}k_{3}[X{]}^{n_{1}}[Y{]}^{n_{2}}}{1+k_{1}[X{]}^{n_{1}}+k_{2}[Y{]}^{n_{2}}+k_{3}[X{]}^{n_{1}}[Y{]}^{n_{2}}}-d_{1}[X]+u_{x}\\ \frac{d[Y]}{dt}=\frac{r_{0}^{y}+r_{4}k_{4}[Y{]}^{n_{2}}+r_{5}k_{5}[X{]}^{n_{1}}+r_{6}k_{6}[X{]}^{n_{1}}[Y{]}^{n_{2}}}{1+k_{4}[Y{]}^{n_{2}}+k_{5}[X{]}^{n_{1}}+k_{6}[X{]}^{n_{1}}[Y{]}^{n_{2}}}-d_{2}[Y]+u_{y}, \end{array}\right.$$

### Stochastic simulations

In Equation 22, parameters of the AND-AND motif (Figure 2C top panel) are: ($r_{0}^{x}$ , $r_{0}^{y}$ , *r_1_*, *r_2_*, *r_3_*, *r_4_*, *r_5_*, *r_6_*) = (0, 0, 0.95, 0, 0, 0.95, 0, 0), (*k_1_*, *k_2_*, *k_3_*, *k_4_*, *k_5_*, *k_6_*) = (1.5, 0.8, 1, 1.5, 0.8, 1) and (*d_1_*, *d_2_*) = (0.55, 0.55). Parameters of the OR-OR motif (Figure 2C bottom panel) are: ($r_{0}^{x}$ , $r_{0}^{y}$ , *r_1_*, *r_2_*, *r_3_*, *r_4_*, *r_5_*, *r_6_*) = (0.15, 0.15, 0.95, 0, 0.95, 0.95, 0, 0.95), (*k_1_*, *k_2_*, *k_3_*, *k_4_*, *k_5_*, *k_6_*) = (2.1, 1.7, 1.8, 2.1, 1.7, 1.8) and (*d_1_*, *d_2_*) = (0.55, 0.55). In both the AND-AND and OR-OR motifs, (*n_1_*, *n_2_*) = (2, 2). We simulated noise in the model using a Langevin equation (Foster et al., 2009):

(27) $$\begin{array}{c} dx_{i}=F_{i}\left(x\right)dt+dW_{i}, \end{array}$$

Where $F_{i}\left(x\right)$ is the deterministic function of Equation 22. $W_{i}$ is a Wiener process which introduces additive noise with no dependence on the state ***x***. We integrated the Wiener process over the interval which gives us $\sqrt{∆t}\xi_{i}$ and $\xi_{i}$ is a Gaussian white noise with zero mean and given variance (*σ^2^*). Thus we can compute the new state of the dynamical systems using the Runge–Kutta method.

### Parameter screening

We conducted global parameters screening of the fully connected stage and Progression-Accuracy trade-off in our models. First, to collect parameter sets with 3 SSSs, we used Latin hypercube sampling (LHS) to screen k-series parameters symmetrically (i.e., *k_1_* = *k_4_*, *k_2_* = *k_5_*, *k_3_* = *k_6_*) ranging from 0.001 to 5 both in the AND-AND and OR-OR motifs. We ultimately collected 6,231 sets for the AND-AND motif and 6682 sets for the OR-OR motifs (Supplementary file 1).

To analyze the sequence of vanishing SSSs. We further filtered parameter sets with 2 SSSs remained as increasing ux (6207 sets for the AND-AND motif; 6634 sets for the OR-OR motif). For each SSS, we quantified the Minus of [X] and [Y] (Figure 5—figure supplement 1C, D).

### Saddle points and saddle dynamics

Set an autonomous dynamical system (Yin et al., 2021)

(28) $$\begin{array}{c} \dot{x}=F\left(x\right),x\in R^{n} \end{array}$$

where $F:R^{n}\to R^{n}\text{ is a }C^{r}\left(r⩾2\right)$ function, and a point $x^{*}\in R^{n}$ is called a stationary point (or equilibrium solution) of Equation 28 if $F\left(x^{*}\right)=0$. Let $J\left(x\right)=\nabla F\left(x\right)$ denote the Jacobian of $F\left(x\right)$ . For a stationary point $x^{*}$ , taking $x=x^{*}+y$ in Equation 28, we have

(29) $$\begin{array}{c} \dot{y}=J\left(x^{*}\right)y+O\left(∥y{∥}^{2}\right), \end{array}$$

where ∥*∥ denotes the norm induced by the inner product. The associated linear system

(30) $$\begin{array}{c} \dot{y}=J\left(x^{*}\right)y, \end{array}$$

is used to determine the stability of $x^{*}$ . Depending on the eigenvalues of $J\left(x\right)$ with positive, negative, and zero real parts, we can define unstable, stable, and center manifolds of the Jacobian $J\left(x\right)$ spanned by the corresponding eigenvectors, as $W^{u}\left(x^{*}\right),W^{s}\left(x^{*}\right)\text{and}W^{c}\left(x^{*}\right)$ .

From the primary decomposition theorem, $R^{n}$ can be decomposed as a direct sum:

(31) $$R^{n}=W^{u}\left(x\right)⊕W^{s}\left(x\right)⊕W^{c}\left(x\right),$$

A hyperbolic stationary point is called a saddle if $W^{u}\left(x^{*}\right)$ and $W^{s}\left(x^{*}\right)$ are nontrivial. The hyperbolic stationary point $x^{*}$ is called a sink (source) if all the eigenvalues of $J\left(x^{*}\right)$ have negative (positive) real parts. The index of a stationary point $x^{*}$ is defined as the dimension of the unstable subspace $W^{u}\left(x^{*}\right)$ .

The HiOSD method (Yin et al., 2019) is designed for finding index-k saddles of an energy function $E\left(x\right)$ . For gradient systems, $F\left(x\right)=-\nabla E\left(x\right)$ , and the Jacobian $J\left(x\right)=-\nabla^{2}E\left(x\right)=-G\left(x\right)$ , where $G\left(x\right)$ denotes the Hessian of $E\left(x\right)$ . The k-saddle $x^{*}$ is a local maximum on the linear manifold $x^{*}+W^{u}\left(x^{*}\right)$ and a local minimum on $x^{*}+W^{s}\left(x^{*}\right)$ . So, the high-index saddle dynamics (HiSD) for a index k saddle (k-saddle) is a transformed gradient flow

(32) $$\begin{array}{c} \dot{x}=-P_{W^{u}\left(x\right)}F\left(x\right)+\left(F\left(x\right)-P_{W^{u}\left(x\right)}F\left(x\right)\right)=\left(I-2P_{W^{u}\left(x\right)}\right)F\left(x\right), \end{array}$$

where $P_{V}$ denotes the orthogonal projection operator on a finite-dimensional subspace V. Here, $-P_{W^{u}\left(x\right)}F\left(x\right)$ is taken as an ascent direction on the subspace $W^{u}\left(x\right)$ and $F\left(x\right)-P_{W^{u}\left(x\right)}F\left(x\right)$ is a descent direction on the subspace $W^{s}\left(x\right)$ . The subspace $W^{u}\left(x\right)=span{\{v}_{1},\ldots ,v_{k}$ **}** where $v_{i}$ is the unit eigenvector corresponding to the smallest i-th eigenvalues, which can be obtained by many methods such as minimize the Rayleigh quotients. According to the above the HiSD for a k-saddle (k-HiSD) is:

(33) $$\left\{ \begin{array}{l} \beta^{-1}\dot{x}=\left(I-\sum_{i=1}^{k}2v_{i}v_{i}^{⊤}\right)F\left(x\right),\\ \gamma^{-1}{\dot{v}}_{i}=-\left(I-v_{i}v_{i}^{⊤}-\sum_{j=1}^{i-1}2v_{j}v_{j}^{⊤}\right)G\left(x\right)v_{i},i=1,2,\ldots ,k, \end{array}\right.$$

which coupled with the initial condition

(34) $$\begin{array}{c} x\left(0\right)=x^{\left(0\right)}\in R^{n},v_{i}\left(0\right)=v_{i}^{\left(0\right)}\in R^{n},\text{s}\text{.}\text{t}\text{.}\left\langle v_{j}^{\left(0\right)},v_{i}^{\left(0\right)}\right\rangle =\delta_{ij},i,j=1,\ldots ,k \end{array}$$

Where I is the identity operator and *β*, *γ*>0 are relaxation parameters.

Similarly, the GHiSD (Yin et al., 2021) for a k-saddle (k-GHiSD) of the dynamical system (28) has the following form:

(35) $$\left\{ \begin{array}{l} \dot{x}=\left(I-2\sum_{j=1}^{k}v_{j}v_{j}^{T}\right)F\left(x\right)\\ {\dot{v}}_{i}=\left(I-v_{i}v_{i}^{T}\right)J\left(x\right)v_{i}-\sum_{j=1}^{i-1}v_{j}v_{j}^{T}\left(J\left(x\right)+J^{T}\left(x\right)\right)v_{i},i=1,\ldots ,k \end{array}\right.$$

which coupled with the initial condition(34). The k-GHiSD can be accelerated by the Heavy Ball method.

### Constructing the solution landscape

For a given set of parameters, we can use k-GHiSD to find each order saddle point of the dynamical system, and then construct the solution landscape of the system. The solution landscape is a pathway map consisting of all stationary points and their connections (Yin et al., 2020; Yin et al., 2022). Solution landscape is a new tool used to describe the dynamic behavior of stationary points in a dynamic system which can show the connection and transfer path between stationary points. In general, we can use the downward search algorithm and the upward search algorithm to construct the solution landscape, which starting from the k-saddle points and the stable points respectively. Because of the symmetry and other prior knowledge of the dynamic system used in our paper, The saddle points of the system tend to occur in the range of ${\left[0,x_{max}\right]}^{2}$ , where the $x_{max}$ means the maximum coordinate of the stable point. We grid the range, select each grid point as the initial state, and find saddle points of corresponding order (*k* = 1,2) using k-GHiSD. Taking the saddle points found before as the initial states, we disturb its unstable directions to search other stationary points of lower index, establish the connection relationship between stationary points and finally construct the solution landscape. In addition, we use geometric minimum action method (gMAM) (Vanden-Eijnden and Heymann, 2008) to find the minimum action path between the stable points, use the obtained action to represent the stability of the stable points, and represent them at different heights in the solution landscape.

### Screening the fully-connected stage

As shown in the case of *u* = 0.0565 in Figure 4D, the fully connected stage (FCS) has three stable points and three 1-index saddle points to connect the stable points with each other. Therefore, we posited that finding a fully connected stage can be equivalent to finding three different 1-index saddle points in this system.

First, build a set of parameters with three stable points. In two different logics, 10,000 sets of dynamic parameters are randomly generated respectively, and all stable points of the system corresponding to each set of parameters are found. If there are three stable points, the corresponding dynamic parameters are recorded as a new set of parameters for the subsequent search of 1-index saddle points.

Second, finding the 1-index saddle points in a system with three stable points. According to the above results, we can find that saddle points tend to appear in the range of ${\left[0,x_{max}\right]}^{2}$ , where the $x_{max}$ means the maximum coordinate of the stable point. So we can grid the range in the same process as before, and search 1-index saddle points with each grid point as the initial structure. In order to find the relevant saddle points as detailed as possible, denser grid points were used as the initial structure of the system without three 1-index saddle points to find the 1-index saddle points.

Due to the calculation error of numerical calculation itself, the saddle points found by saddle point dynamics may have the following problems:

1. The same saddle points found from different initial structures were mistaken for two.
2. Find a negative or infinite saddle point.
3. The saddle-point judgment is inaccurate due to the influence of zero eigenvalue.

We can set corresponding judgment conditions to solve the above problems.

In addition, since there is also a parameter *u* representing the foreign signal in the system, we can let *u* vary to find if there are three 1-index saddle points. At the beginning, *u* = 0 is set, and then *u* is changed according to the number of saddle points found and the corresponding judgment conditions, so as to find the system with three 1-index saddle points. We use the idea of adaptive step size to speed up the search process in the algorithm of finding three 1-index saddle points in the system. The specific process is as follows:

- Set the initial u = 0, du = 1.
- Saddle Dynamics is used to find the 1-index saddle points in the parameters.
- When the number of 1-index saddle points is equal to 0: there is only one stable point in the system (*u* = 0.12 in Figure 4F), it indicates that u is too large and needs to be reduced to make *u* = *u* du.
- When the number of 1-index saddle points is equal to 1: the system has two stable points (*u*=0.12 in Figure 4C), it indicates that u is too large and needs to be reduced so that *u* = *u* du.
- When the number of 1-index saddle points is equal to 2: the system has two kinds of cases, one of which needs to make *u* increase (*u* = 0 in Figure 4C). In this case, the coordinate of stable point near *y* = *x* is smaller than the midpoint of two 1-index saddle points, so we let *u* = *u +* du, and let du = 0.1 * du. In the other case, there are two possibilities. One is like *u* = 0 in Figure 4.F, and the other is like *u* = 0.0595 in Figure 4F. In this case, there is no stable point near *y* = *x* or the coordinate of the stable point is larger than the midpoint of two 1-index saddle points.
- When the system finds that the number of 1-index saddle points is equal to 3: we need to judge whether there are three non-degenerate saddle points that are different from each other. If the condition is sufficient, it indicates that we have found the fully connected solution landscape; otherwise, let *u* = *u +* du, du = 0.1 * *du*, and continue searching.
- When the number of 1-index saddle points is greater than 3, it indicates that *u* is too large and needs to be reduced to make *u* = *u* du.
- If three 1-index saddle points are not found after 100 changes of *u*, it is considered that there is no fully connected solution landscape in the system.

We, respectively, used the above algorithm to search for the fully connected solution landscape under two different logics and obtained the following results:

**Table:** 

Logic	# of 3 SSS	# of FCS	Ratio
AND-AND	6231	5151	82.7%
OR-OR	6682	120	1.8%

It can be seen from the simulation results that the fully connected solution landscape can appear in most AND-AND motifs, but almost not in the OR-OR motifs. Due to the error of numerical calculation itself, the obtained results may have a certain deviation. In addition, in our search process, due to the limitation of computing resources and the complex nature of some systems, in order to take into account the majority of cases, some systems with full connectivity have not been found. Subsequent manual search found that the fully connected solution landscape is existed in some systems which not found under the AND-AND motifs previously (17.3%). Therefore, the obtained proportion can be further improved.

### Data processing of single-cell data

There are totally four public datasets used in our work (related to Figure 6F and H and Figure 7 and Figure 6—figure supplement 2C). In mice’s hematopoiesis (Mojtahedi et al., 2016), expression of genes is quantified as 2^LOD−Cq^−1 (LOD: limit of detection; Cq-value is the cutoff of amplification signal in single-cell qPCR data). Thus, we can compute the coefficient of variation (CV) over time. In embryogenesis (Semrau et al., 2017), public dataset used in our work was generated by single-cell SMART-seq2 (GEO: GSE79578). We utilized preprocessed data to compute the CV over time. In reprogramming, public dataset was generated by 10 x Genomics (GEO: GSE207654). Counts Per Million (CPM) matrix are used to compute the CV. We assigned time points by the Leiden clustering algorithm with parameters consistent with the original paper (see methods in Qin et al., 2022).

### Fuzzy C-means clustering

CV of 2000 high variable genes given by original paper (Qin et al., 2022) over four time points were computed. We filtered out 1677 genes by removing NA due to missing values. Noise of 1677 genes were then grouped into 12 clusters using the Mfuzz package in R with fuzzy c-means algorithm (Kumar and Futschik, 2007; Supplementary file 2).

### GO enrichment analysis

GO and pathway enrichment analyses were performed using Metascape (Zhou et al., 2019).

### Integration of human TF-target interactions

A collection of 456,698 TF-target interactions for 1541 human TFs and their mode of regulation (MoR, activation or repression) were integrated by merging a built-in collection of human regulons from the DoRothEA R package (version 1.8.0) (Garcia-Alonso et al., 2019) and human TRRUST database (Han et al., 2018). To be specific, resource from DoRothEA includes 454,504 TF-target interactions for 1541 human TFs, retrieved from (1) literature-curated resources, (2) ChIP-seq binding data, (3) TFBS (TF binding sites) predictions, and (4) transcriptional regulatory interactions inferred from published gene expression profiles. Human TRRUST database is a manually curated database of transcriptional regulatory networks, whose current version contains 8427 TF-target regulatory relationships of 795 human TFs with annotation of MoR (activation, repression, or unknown). For our purpose, we excluded TF-target interactions with unknown MoR, yet 207 TF-target pairs still remained to show conflict MoRs in records from literature, according to TRRUST database. To integrate data from both resources, when a conflict occurred in TRRUST database, we retained the MoR recorded in DoRothEA resource for the same pair of TF-target, but omitted the conflict records if the TF-target pair was not included in DoRothEA resource. As for TF-target pairs without conflict MoRs in TRRUST database but shows conflicts across the two resources, records in TRRUST database were taken as more credible (Supplementary file 3).

### Integration of human TF list

In addition to the 1541 human TFs we obtained from the collection of TF-target interactions mentioned earlier, 1639 human TFs reported by Lambert et al., 2018 and 1564 by Ng et al., 2021 were used as complements, ending up with a total of 2051 human TFs (Supplementary file 3).

## Data Availability

All data analysed during this study are included in the manuscript and supporting files. The following previously published datasets were used: SemrauS van OudenaardenA 2017Dynamics of lineage commitment revealed by single-cell transcriptomics of differentiating embryonic stem cellsNCBI Gene Expression OmnibusGSE7957810.1038/s41467-017-01076-4PMC565365929061959 QinJ ZhangJ JiangJ LiY PeiX 2022Direct chemical reprogramming of human cord blood erythroblasts to induced megakaryocytes that produce plateletsNCBI Gene Expression OmnibusGSE20765410.1016/j.stem.2022.07.00435931032

## Additional files

Supplementary file 1. Parameter sets for screening fully-connected stage in two logic motis. The spreadsheet titled ‘MAP’ gives the relative energy in Figure 4D and G, Figure 5E and G, Figure 7D.

Supplementary file 2. 1677 TFs divided into 12 clusters based on time-course CV. The spreadsheet lists all 12 clusters of TFs, as described in Figure 7F, Figure 7—figure supplement 1C.

Supplementary file 3. Integrated human TF list and regulon database. The spreadsheet records the human TF list and regulon used in Figure 7H.

MDAR checklist
