# Supplementary material for: A logic-incorporated gene regulatory network deciphers principles in cell fate decisions
Source: eLife. 2024 Apr 23;12:RP88742. doi: 10.7554/eLife.88742 (PMC11037919; doi:10.7554/eLife.88742)
Supplement: Peer review history

# Editor report: eLife assessment

**DOI:** 10.7554/eLife.88742.3.sa0

**Authors:** Mariana Gómez-Schiavon

**Affiliations:** Universidad Nacional Autónoma de México Mexico

**Keywords:** Important, Solid

The study presented in this manuscript makes **important** contributions to our understanding of cell fate decisions and the role of noise in gene regulatory networks. Through computational and theoretical analysis, the authors provide **solid** support for distinguishing distinct driving forces behind fate decisions based on noise profiles and reprogramming trajectories. While acknowledging the potential limitations of small gene regulatory networks in capturing the richness of whole-transcriptome sequencing datasets, this study offers a creative approach for formulating hypotheses about gene regulation during stem cell differentiation using single-cell sequencing data.

# Referee report: Joint public review

**DOI:** 10.7554/eLife.88742.3.sa1

In this manuscript, Xue and colleagues investigate the fundamental aspects of cellular fate decisions and differentiation, focusing on the dynamic behaviour of gene regulatory networks. It explores the debate between static (noise-driven) and dynamic (signal-driven) perspectives within Waddington's epigenetic landscape, highlighting the essential role of gene regulatory networks in this process. The authors propose an integrated analysis of fate-decision modes and gene regulatory networks, using the Cross-Inhibition with Self-activation (CIS) network as a model. Through mathematical modelling, they differentiate two logic modes and their effect on cell fate decisions: requires both the presence of an activator and absence of a repressor (AA configuration) with one where transcription occurs as long the repressor is not the only species on the promoter (OO configuration).

The authors establish a relationship between noise profiles, logic-motifs, and fate-decision modes, showing that defining any two of these properties allows the inference of the third. They also identify, under the signal-driven mode, two fundamental patterns of cell fate decisions: either prioritising progression or accuracy in the differentiation process. The authors apply this analysis to available high-throughput datasets of cell fate decisions in hematopoiesis and embryogenesis, proposing the underlying driving force in each case and utilising the observed noise patterns to nominate key regulators.

The paper significantly advances our understanding of gene regulatory networks through a well-described computational study, where the authors rigorously evaluate assumptions in modelling. Particularly commendable is their introduction of the concept of combinatorial logic, exemplified by the double 'and' and double 'or' (AA/OO) logic motifs, which they successfully map to previously described cell fate decision processes. This theoretical and computational exploration sheds light on the dynamic landscape of epigenetic cell fate decisions, emphasising the role of combinatorial logic in coordinating noise and signal-driven processes. The thorough comparison of two model configurations underscores the importance of integration logic, contributing to a clearer understanding of gene regulatory network dynamics. Importantly, the results of the simulations are presented clearly, enhancing accessibility and intuitive understanding. The paper's strength also lies in its predictive power, as the authors use simulations to make insightful predictions about the regulatory organisation of stem cell differentiation systems. While the exploration is restricted to specific scenarios, these limitations serve to highlight areas for future research rather than detract from the paper's strengths.

While the paper presents an intriguing framework for understanding gene regulatory networks and cell fate decisions, there are some weaknesses that warrant attention. Firstly, the framework would benefit from validation with more experimental data and application to diverse systems beyond those explored in the study, such as de-differentiation in adult tissues and regeneration processes. Additionally, while the authors successfully make predictions about the regulatory organisation of stem cell differentiation systems, there is a lack of discussion regarding how perturbations in the regulatory network could affect cell fate decisions. Furthermore, the paper could be strengthened by addressing the effects of mutations and other perturbations that may significantly influence cell fate decision-making processes, thus enhancing the robustness of the findings. Finally, there are instances where the clarity of the writing could be improved to enhance understanding and accessibility for readers.

# Author comment: Author response

**DOI:** 10.7554/eLife.88742.3.sa2

**Authors:** Gang Xue, Xiaoyi Zhang, Wanqi Li, Lu Zhang, Zongxu Zhang, Xiaolin Zhou, Di Zhang, Lei Zhang, Zhiyuan Li

**Affiliations:** Peking University Beijing China; Peking University Beijing China; Peking University Beijing China; Peking University Beijing China; Peking University Beijing China; Peking University Beijing China; Peking University Beijing China; Peking University Beijing China; Peking University Beijing China

The following is the authors’ response to the original reviews.

We greatly appreciate the editor and reviewers’ careful and professional assessment of this manuscript. We are delighted with the reviewers’ instructive comments and suggestions. We have tried to address the raised points comprehensively. The reviewers’ scrutiny has helped us immensely to discuss and present our work extensively and properly. We are grateful for the reviewers’ efforts and insights. The detailed responses are listed here.

**Recommendations for the authors**

(1) The intuition behind the model is not properly explained, i.e., the derivation of Eqs. 1-2 and the biological meaning of the AA/OO logic modes. A different notation could be helpful.

We thank the reviewers for this comment, and agree that the interpretation of our model in manuscript was indeed in need of improvement. We have incorporated this suggestion into the manuscript. For clarity, we have substituted AND-AND/OR-OR for original expression of AA/OO, and hope that new notations are helpful for interpreting our work.

In general, considering the diverse audience including those with experimental background, we feel that it is essential to present this manuscript in a more digestible manner. We therefore retain the entire derivation of Eqs. 1-2 in the supplementary method. We have added a qualitative introduction to model derivation and molecular biological significance underlying different logic motifs (AND-AND/OR-OR) in the revised manuscript. Please refer to Page 5 of the revised manuscript, lines 161-167 (see below).

$$\frac{d[Y]}{dt}=\frac{r_{0}^{y}+r_{4}k_{4}[Y{]}^{n_{2}}+r_{5}k_{5}[X{]}^{n_{1}}+r_{6}k_{6}[X{]}^{n_{1}}[Y{]}^{n_{2}}}{1+k_{4}[Y{]}^{n_{2}}+k_{5}[X{]}^{n_{1}}+k_{6}[X{]}^{n_{1}}[Y{]}^{n_{2}}}-d_{2}[Y]$$

“X and Y are TFs in the CIS network. n1 and n2 are the coefficients of molecular cooperation. k1-k3 in Eq1 and k4-k6 in Ep2 represent the relative probabilities for possible configurations of binding of TFs and CREs. (Fig2.A). d1 and d2 are degradation rates of X and Y, respectively. Here, we considered a total of four CRE’s configurations as shown in Figure 2A (i.e., TFs bind to the corresponding CREs or not, 2^2=4). Accordingly, depending on the transcription rates (i.e., r0x, r1, r2, r3 in Eq1, similarly in Eq2) of each configuration, we can model the dynamics of TFs in the Shea-Ackers formalism[1, 2].

Thus, the distinct logic operations (AND/OR) of two inputs (e.g., activation by X itself and inhibition by Y) can be further implemented by assigning corresponding profile of transcription rates in four configurations (Fig2.A). From the perspective of molecular biology, the regulatory logics embody the complicated nature of TF regulation that TFs function in a context-dependent manner. Considering the CIS network, when X and Y bind respective CREs concurrently, whether the expression of target gene is turned on or off depends on the different regulatory logics (specifically, off in the AND logic and on in the OR logic; Fig2.A). Notably, instead of exploring the different logics of one certain gene[3, 4], we focus on different combinations of regulatory logics due to dynamics in cell fate decisions is generally orchestrated by GRN with multiple TFs.”

(2) More clearly specify the used parameters and how these are chosen. This would be helpful to get a more quantitative grasp of the conditions that they compare.

We appreciate the reviewers pointing out unspecified parts in the main text. We have now included related discussion in the revised manuscript. Please refer to Page 5 of the revised manuscript, lines 179-181 (“Benchmarking the Boolean models with different logic motifs (Fig2.B), we reproduced the geometry of the attractor basin in the continuous models resembling those represented by corresponding Boolean models (Fig2.C; see Methods).”).

We would like to highlight that the Boolean models with different logic motifs (Fig. 2B) explicitly display the difference of state spaces (i.e., attractor basin). Moreover, as the focus of this work is on the role of regulatory logics in cell fate decisions, we ponder that it is rational to specify the geometry of the landscape based on the hint from Boolean models. Therefore, we reason that it is intuitive and reliable to assign values to used parameters by mapping our ODE models (Eqs. 1-2) to corresponding Boolean models qualitatively (refer to the statement in our original manuscript, Page 5, lines 162-163, “With appropriate parameters, we are able to reproduce the Boolean-like attractor basin in the continuous models”). In producing Figure 2-5, setting of parameters was performed in a heuristic way without particular searching. However, to draw general conclusions, like the "trade-offs between progression and accuracy" and the presence of the fully-connected stage, we sampled a substantial number of sets parameters to ensure statistically robust findings.

(3) Include the explanation of how the nullclines and basins shown in the figures (e.g., Fig. 2C, Fig. 4C, Fig. 4F, etc.) are calculated.

We thank the reviewers for this suggestion. We have incorporated this into the legend of corresponding figures when first mentioned in the main text. Please refer to Page 7 of the revised manuscript, lines 217-223 (see below).

“Fig2.C:

(C) State spaces of the AND-AND (top panel) and OR-OR (bottom panel) motifs in ODE models. Dark and red lines represent nullclines of $\frac{d[X]}{dt}=0,\cdot \frac{d[Y]}{dt}=0$ respectively. Stable steady states (SSS) are denoted as orange dots. Unstable Steady States (USSs) are denoted as white dots. Each axis represents the concentration of each transcription factor, which units are arbitrary. Blue, green and purple areas in state spaces indicate attractor basins representing LX, S and LY, respectively. Color of each point in state space was assigned by the attractors they finally enter according to the deterministic models (Eq1, Eq2). These annotations were used for the following Figure 3-7.”

(4) Clarity on the decisions in the work is needed. For example, the "introduction" of asymmetry of the noise levels (as stated in line 215) appears completely arbitrary. The reason behind it can be guessed in the following paragraph, but the reader shouldn't have to guess.

We agree entirely with the reviewers’ comment. Indeed, this should have been stated more explicitly. The motivation for incorporating asymmetry in the noise levels stems from our endeavor to mimic the inherent biological variability in gene expression within a cell population. We have adjusted the manuscript to better convey the motivation for investigating asymmetric noise level. Please refer to Page 8 of the revised manuscript, lines 237-238 (“In biological systems, it is unlikely that the noise level of different genes is kept perfectly the same.”).

(5) Arbitrary and/or out-of-context jargon is used throughout the manuscript, making it hard to read and follow what the authors mean in some cases. For example, "temporal fully-connected stage" is used for the first time in line 290, and the term is not explained either in the main text or in the manuscript. Similarly, the reference to a Boolean-like and Boolean model (line 163 and Figure 1) without clarifying if this is just an analogy or if a formal model is built, nor the utility and implications of this comparison. Another problem related to jargon occurs on line 291, where the authors talk about "parameter sensibility", but such analysis (as it is normally understood in the field) is never performed; the authors perform a parameter exploration and make some general conclusions about the parameter space, but that is different than a parameter sensitivity analysis.

We thank the reviewers for this comment, as it has prompted us to better clarify our manuscript. We have reviewed the manuscript and made the necessary adjustments to improve its clarity. We do hope that this revision meets the reviewers’ expectations on the clarity and comprehensiveness of our analysis.

Regarding the jargon of "temporal fully-connected stage", we realized that this term was slightly vague and in need of improvement. Instead, we now employ “transitory fully-connected stage” in the revised manuscript to underline the short emergence of this particular stage. Please refer to Page 11 of the revised manuscript, lines 323.

We thank the reviewers for pointing out the lack of clarity concerning the Boolean models. We have now amended the manuscript to make this implicit expression explicit. Please refer to Page 5 of the revised manuscript, lines 179-181 (“Benchmarking the Boolean models with different logic motifs (Fig2.B; see Methods), we reproduced the geometry of the attractor basin in the continuous models resembling those represented by corresponding Boolean models (Fig2.C; see Methods).”). Specifically, we employed the Boolean models (Fig.2B) as the reference to assist us to heuristically evaluate the applicability of used parameters in the ODE models. Therefore, the Boolean models are built formally, and corresponding updated rules are listed in Fig.2A (refer to the middle row in the table called “Logic Function”, now also noted in the legend of Fig.2B, Page 7, lines 213-214). Nevertheless, we do utilize the analogy between the attractor basins from Boolean models and ODE models (refer to Fig.2B-C). Accordingly, we used the term “Boolean-like” to describe the landscape presented by the continuous models (Eqs. 1-2; refer to the statement in our original manuscript, Page 5, lines 162-163, “With appropriate parameters, we are able to reproduce the Boolean-like attractor basin in the continuous models”).

We appreciate the reviewers for this valuable comment, and agree that the usage of “parameter sensibility” was in need of adjustment. We have now amended the manuscript. Please refer to Page 10 of the revised manuscript, lines 318-321 (see below).

“To manifest the generality, we globally screened 6,213 groups of parameter sets under the AND-AND motif, and this logic-dependent intermediated stage can be observed for 82.7% of them (see Methods; Table S1), indicating little dependence on particular parameter setting (1.8% in the OR-OR motif).”

(6) Probably related just to the language clarity (i.e., the abuse of jargon), but we don't understand the conclusion on lines 296-298.

We thank the reviewers for this comment. We have adjusted the manuscript accordingly. Please refer to Page 11 of the revised manuscript, lines 323-327 (see below). And we hope that the reviewers agree with our attempt at mapping into the particular stage in cell fate decisions from the point of landscape.

“Furthermore, this transitory fully-connected stage locates between the fate-undetermined stage (Fig4.C top panel) and fate-determined stage (Fig4.C 3rd panel), comparable to the initiation (or activation) stage before the lineage commitment in experimental observations [5-7]. Therefore, we suspected that the robust fully-connected stage in the AND-AND motif may correspond to a specific period in cell fate decisions.”

(7) The so-called "solution landscape" in Figure 4E needs to be better explained.

We thank the reviewers for this comment. We have introduced the concept of solution landscape, which is a pathway map consisting of all stationary points and their connections, in lines 196-198 of the revised manuscript (see below).

“Furthermore, we introduced the solution landscape method. Solution landscape is a pathway map consisting of all stationary points and their connections, which can describe different cell states and transfer paths of them [82-84].”

In Figure 4E, we added detailed explanation of the solution landscape for the AND-AND motif. Specifically, it describes a hierarchical structure including one 2-saddle (yellow triangle), three 1-saddles (crimson X-cross sign), and three attractors (green dot). The layer of 1-saddles is represented by a blue translucent plane, and the bottom layer is the flow field diagram. The connections from 2-saddle to 1-saddles and from 1-saddles to the attractors are represented by red and blue lines, respectively. The arrow and color of the heatmap correspond to the flow direction and the length of the acceleration at each point in the state space.

(8) Table S1 is not properly annotated, and then it is impossible to interpret how it supports the observations in the paragraph in lines 342-342.

We appreciate the reviewers’ useful feedback. We have refined the annotations of all tables in our manuscript (Table S1-3). Please refer to “Supplementary Table” in resubmitted files.

Specifically, we randomly collected 6,231 sets of parameters for the AND-AND motif and 6,682 sets for the OR-OR motif (k1-k6 in Eq1 and Eq2; refer to Page 6 of the revised supplementary method, see below).

“First, to collect parameter sets with 3 SSSs, we used Latin hypercube sampling (LHS) to screen k-series parameters symmetrically (i.e., k1 = k4, k2 = k5, k3 = k6) ranging from 0.001 to 5 both in the AND-AND and OR-OR motifs. We ultimately collected 6,231 sets for the AND-AND motif and 6,682 sets for the OR-OR motifs (Table S1).”

To analyze the sequence of vanishing SSSs, we further filtered parameter sets with 2 SSSs remained as increasing ux (corresponding to Eq3 in the revised manuscript, Page 10, lines 293). We then got a collection of 6,207 sets for the AND-AND motif and 6,634 sets for the OR-OR motif. Based on these parameter settings, we checked if the observations (refer to Page 13, lines 377-378, “The distinct sequences of attractor basin disappearance as ux increasing can be viewed as a trade-off between progression and accuracy.”) are artifacts of particular parameter choice.

(9) The flow in Section 5 needs to be reorganised. For instance, it is not clear which question the authors are addressing in line 395, or how the proposed approach answers the question stated in lines 381-382.

We greatly thank the reviewers for pointing this out, and acknowledge that the Section 5 was definitely in need of improvement. We have now amended the manuscript to make this implicit understanding explicit. Please refer to Page 15 of the revised manuscript, lines 426-430 (see below).

“In prior sections, we systematically investigated two logic motifs under the noise- and signal-driven modes in silico. With various combinations of logic motifs and driving forces, features about fate-decision behaviors were characterized by computational models. Next, we questioned whether observations in computation can be mapped into real biological systems. And how to discern different logic motifs and driving modes is a prerequisite for answering this question.

To end this, we first evaluated the performance of different models, specifically in simulating the process of stem cells differentiating towards LX (Fig6.A).”

(10) There are two important weak points for the successful classification of the regulatory logic of real gene expression data as presented in the manuscript: (1) the small number of time-points in the datasets and clear peaks in gene expression heterogeneity cannot be identified, and (2) it is not always clear whether cell differentiation really exclusively relies on a CIS network, and which genes constitute it. These limitations should be solved or at least discussed in the manuscript.

We thank the reviewer for this comment. First, we agree entirely that analysis of datasets with more time points will be more amenable to identifying the trends of gene expression variation. We have made a concerted effort towards searching for such datasets, but unfortunately, there are not many such datasets publicly available. Specifically, to apply our computational framework, the datasets of our interest need to fulfill the following three characteristics: (i) sampling at multiple time points (as many as possible); (ii) to illustrate/validate our findings clearly and representatively, we would like the cell fate decisions in the biological systems to follow the classical binary tree-like pattern. i.e., there is one stem cell fate (or progenitor) and two downstream cell fates in the systems; (iii) the core GRN circuits for orchestrating the fate-decision processes have been experimentally confirmed (at least clearly supported). We have also extended the discussion to include above points to explicitly note the limitations regarding the used datasets. Please refer to Page 25 of the revised manuscript, lines 762-766 (see below).

“The gene expression datasets analyzed here are only available for a limited number of time points. Though they meet the need for discerning trends, it is evident that the application to the datasets with more time points will yield clearer and less ambiguous changing trends to support the conclusions of this paper more generally.”

In regards to second point, we do acknowledge that the CIS network may not always be the core module for every fate-decision case (but to our knowledge, this can be assumed in many cases, especially in binary tree-like pattern). For applicability and potential relevance to our intended readership, we developed the models and draw our conclusions primarily based on the CIS topology for its representativeness. We intend to incorporate diverse topologies (like mutual activation with self-activation, Feed-Forward Loop, etc.) in our computational framework presented here in near future. Additionally, we have incorporated this point into the discussion in the revised manuscript. Please refer to Page 25 of the revised manuscript, lines 766-769 (see below).

“Notwithstanding the fact that the CIS network is prevalent in fate-decision programs, there are other topologies of networks that serve important roles in the cell-state transitions, like feed-forward loop, etc. The framework presented in this work should further incorporate diverse network motifs in the future.”

As referred by the reviewers, even if given the CIS network, we may not sure about which genes constitute it in some cases. We agree that further extension of our framework to mining key regulators is an interesting question. We also note that we have become very enthusiastic about recent work that shows how to nominate core factors from high-throughput data[8, 9]. Of note, in the last section of our manuscript titled “The chemical-induced reprogramming of human erythroblasts (EBs) to induced megakaryocytes (iMKs) is the signal-driven fate decisions with an OR-OR-like motif”, we leveraged patterns of temporal expression variance to filter out key regulators (Fig7.F and H). We thus underline the potential of mining genes comprising core GRN circuits through expression variance. Nevertheless, as the focus of the present paper is on the role of regulatory logic in cell fate decisions, we feel it is beyond the scope of the present article to continue the development of our results on this point. Instead, we have included discussion of case that genes comprising the CIS network are not defined. Please refer to Page 23 of the revised manuscript, lines 685-687 (see below).

“Notably, if the genes that constituting the CIS network are not specified, we can conversely leverage the patterns of temporal expression variance to nominate key regulators in a model-guided manner.”

(11) The models used in Figure S5 are never clearly described.

We thank the reviewers for pointing this out. We have now introduced the settings of the models used in Figure S5 more clearly in the legend (see below).

Two logic motifs with the noise-driven mode (FigS5.A, see below):

**Author response image 1.** Initial values were identical with attractor of S fate in Figure 2C (SSSs in green attractor basins). Simulation was preformed 1000 times for each pseudo-time point, with each temporal state (from left to right) recorded as a dot on the plot. Top panel: Noise level of X (σx) is set to 0.21, and σy is 0.09. Bottom panel: Noise level of Y (σy) is set to 0.21, and σx is 0.09. Red arrow represents the direction of fate transitions of S to LX. Other than adding a white noise, parameters were identical with those in Figure 2C.”

Two logic motifs with the signal-driven mode (FigS5.B, see below):

**Author response image 2.** Initial values were identical with attractor of S fate in Figure 2C (SSSs in green attractor basins). Top panel: Noise level of X (σx) and Y (σy) are both set to 0.06. Simulation was preformed 1000 times, with each final state recorded as a dot on the plot. Parameter ux switched from 0 to 0.09 (0, 0.045, 0.09, from left to right). Bottom panel: Noise level of X (σx) and Y (σy) are both set to 0.05. Simulation was preformed 1000 times, with each final state recorded as a dot on the plot. Parameter ux switched from 0 to 0.24 (0, 0.12, 0.24, from left to right). Red arrow represents the direction of fate transitions of S to LX. Other model’s parameters were identical with those in Figure 2C.”

(12) Up until Section 5, "noise levels" have been used to refer to an input/parameter in the model. Here it is assumed as an emergent property. Are the authors talking about the variance in expression (e.g., see line 398)? Is it defined as the coefficient of variation? Clarity is essential to interpret the observations in this section, e.g., "different driving modes change in the patterns of noise rather than expression levels" (lines 399-400).

We greatly appreciate the reviewers pointing this ambiguity out. The term of “noise level” was indeed used to refer the strength of the noise in the models in Section 1-4. For classifying different logic motifs with two driving forces, we needed a practical metric that can be quantified from data, and we found population-level gene expression variance (i.e., “noise level” in line 398) is useful which defined as the coefficient of variation. For clarity, we carefully decide to substitute “expression variance” for “noise level” presented in Section 5-6. We have amended the manuscript accordingly, and hope this revision will be helpful for interpreting our result. Please refer to Page 15 of the revised manuscript.

(13) "Pulse-like behaviour" is used in an arbitrary way, not as it is normally used in the field. Moreover, we consider this jargon expression does not contribute to the understanding of the paper. (The authors probably meant "discrete transitions" vs "gradual transitions".)

We appreciate the reviewers’ valuable feedback regarding our use of the term “Pulse-like behavior”. We agree with the reviewers’ statement, and acknowledge that terminology of noise level’s patterns between different driving modes (noise-driven vs signal-driven; refer to Section 5 in our manuscript) was in need of improvement.

Upon comprehensive consideration, we primarily decided to adopt the terms “monotonic transitions” and “nonmonotonic transitions” to recapitulate the trends of noise level, underlining the distinct temporal noise’s patterns in cell fate decisions brought by two driving forces in a more contrastive way. We anticipate that current jargon expressions will be beneficial for interpreting our work. Please refer to Page 15 of the revised manuscript.

(14) The temporal resolution of the scRNAseq datasets that the authors used is too low to unambiguously distinguish a discrete pattern of gene expression heterogeneity from a rising profile. This limitation needs to be at least acknowledged in the text. Alternatively, the authors might want to identify more recent datasets with higher time resolution.

We appreciate the reviewers’ insightful suggestions. We agree that analysis of datasets with higher time resolution will be more unambiguous to identifying the trends of gene expression variation. We have made a concerted effort towards searching for such datasets, but unfortunately, there are not many such datasets publicly available. Specifically, to apply our computational framework, the datasets of our interest need to fulfill the following three characteristics: (i) sampling at multiple time points (as many as possible); (ii) to illustrate/validate our findings clearly and representatively, we would like the cell fate decisions in the biological systems to follow the classical binary tree-like pattern. i.e., there is one stem cell fate (or progenitor) and two downstream cell fates in the systems; (iii) the core GRN circuits for orchestrating the fate-decision processes have been experimentally confirmed (at least clearly supported). Nevertheless, we recognize this limitation should be mentioned in the paper. So, we have also extended the discussion to include above points. Please refer to Page 25 of the revised manuscript, lines 762-766 (see below).

“The gene expression datasets analyzed here are only available for a limited number of time points. Though they meet the need for discerning trends, it is evident that the application to the datasets with more time points will yield clearer and less ambiguous changing trends to support the conclusions of this paper more generally.”

(15) In the case of embryonic stem cell differentiation, an additional complication is that this protocol yields heterogeneous cell type mixtures, whereas the authors' simulations usually are designed to give differentiation towards a single cell type. This difference makes it difficult to compare measures of gene expression heterogeneity between simulations and the experimental system to infer regulatory logic questionable.

We thank the reviewers for this valuable comment and realize that we were not clear enough in the manuscript regarding the case of embryogenesis. In the biological system devised by Semrau et al[10], mouse embryonic stem cells (mESCs) differentiates into two lineages simultaneously, just as mentioned by the reviewers. We noticed this additional complication and performed other simulations in two logic motifs with increasing noise level of gene X and Y, as presented in Fig.S6E (see below).

**Author response image 3.** 

“(E) Time courses on the coefficient of variation in expression levels of X and Y genes in silico during differentiation under the noise-driven mode. Initial values were set to the attractors of S fate in Figure 2C (SSSs in green attractor basins). Top panel: Noise level of X (σx) and Y (σy) are both set to 0.14. Bottom panel: Noise level of X (σx) and Y (σy) are both set to 0.1. Stochastic simulation was preformed 1000 times for each pseudo-time point.”

Given the noise-driven mode, we further employed the expression pattern of Gbx2-Tbx3 circuit to heuristically infer the logic motif.

(16) In contrast to the hematopoiesis example, the authors do not focus on a specific gene regulatory circuit with the ESC dataset. How their approach is possible on genome-wide data needs to be discussed.

We thank the reviewers for this comment. Indeed, the core GRN orchestrating the fate-decision process reported by Semrau et al[10] is not fully elucidated. We here focus on the Gbx2-Tbx3 circuit (Fig.6H, Fig.S6D). These two TFs were filtered out from 22 candidate TFs and suggested as potential key regulators in the original paper[10]. Accordingly, at this point we followed the original paper’s statement.

In regards to extension into biological systems without specific gene regulatory circuits, we have included discussions about the possibility that genes comprising the CIS network are not defined. Please refer to Page 23 of the revised manuscript, lines 685-687 (see below).

“Notably, if the genes that constituting the CIS network are not specified, we can conversely leverage the patterns of temporal expression variance to nominate key regulators in a model-guided manner.”

(17) [In supplemental material, pp.1] Possible typo: "In our word, we considered a GRN comprised...".

Thanks for spotting this typo. We have amended it in the revised supplemental method (refer to Page 1 of the revised supplementary method).

(18) [In supplemental material, pp.1] In Eqs. (1), the notation for the function HX([X]) implies that HX only depends on X, leaving the combinatorial regulation out. HX([X],[Y]) would be more general and accurate.

Thanks for pointing this out. We have incorporated this suggestion into the manuscript. Please refer to Page 1 of the revised supplementary method.

(19) [In supplemental material, pp.1] There are several works that have shown that the Hill coefficient is rarely representative of the number of binding elements. The model can be more general. See, for example, «Santillán, Moisés. "On the Use of the Hill Functions in Mathematical Models of Gene Regulatory Networks." Mathematical Modelling of Natural Phenomena 3, no. 2 (October 22, 2008): 85-97. https://doi.org/10.1051/mmnp:2008056.» and «Nam, Kee-Myoung, Rosa Martinez-Corral, and Jeremy Gunawardena. "The Linear Framework: Using Graph Theory to Reveal the Algebra and Thermodynamics of Biomolecular Systems." Interface Focus 12, no. 4 (June 10, 2022): 20220013. https://doi.org/10.1098/rsfs.2022.0013.»;

We thank the reviewer for drawing our attention to this and highlighting the above works. Indeed, this is important information to include in the manuscript. We have incorporated this suggestion into the revised supplemental method (refer to Page 1 of the revised supplementary method). These references have now been included in the revised supplemental method (refer to references [2]-[3]).

(20) [Minor] The configuration labels can be confusing, especially the AA, which is rather an AND NOT gate.

We thank the reviewers for this comment. For clarity, we have substituted AND-AND/OR-OR for original expression of AA/OO, and hope that new notations are helpful for interpreting our work.

(21) [Minor] Very low printing quality in Figure 1.

Thanks for the feedback regarding the printing quality of Figure 1. We have made the necessary adjustments to improve its quality. We have also ensured that all other figures in the manuscript meet the required standards.

(22) [Minor] We suggest including a quantitative scale for the bias in Fig. 3E.

Thanks, we have incorporated this suggestion into the manuscript.

(23) [Recommendation] Authors could also evaluate the cell fate decision processes as mutations or other perturbations affect a regulatory network.

We appreciate the reviewers for this valuable recommendation. We agree with the reviewers that further involving new cases would be helpful, especially those mutation-driven disease-related fate-decision processes, such as neutropenia in chemotherapy. However, given the considerable effort towards searching for appropriate datasets, we carefully decide not to make this change.

(24) [Recommendation] The authors could include some discussion of the likely impact of the work on the field and the utility of the methods and data to the community. For example, understanding the fluidity of the epigenetic landscape and the regulatory forces behind cell fate decisions can be of great importance in designing synthetic gene regulatory circuits.

We greatly appreciate the reviewers pointing this out. In the original manuscript, we intentionally limited the length of the discussion to make the whole story more focus. We thank the reviewers for their insightful suggestions regarding the content of discussion. We have incorporated this suggestion into the revised manuscript. Please refer to Page 25, lines 751-757 (see below).

“Recently, synthetic biology has realized the insertion of the CIS network in mammalian cells. One of the prerequisites for recapitulating the complex dynamics of fate transitions in synthetic biology is systematical understanding of the role of GRNs and driving forces in differentiation. And the logic motifs are the essential and indispensable elements in GRNs. Our work also provides a blueprint for designing logic motifs with particular functions. We are also interested in validating the conclusions drawn from our models in a synthetic biology system.”

In addition, a longstanding question of our interest in cell fate decisions is what contributes the distinctive development cross species, like human, mice and so on forth. However, in addition to protein coding sequences, regulatory interactions between genes (i.e., activation and inhibition) also exhibit conservation as reported in recent work of multi-species cell atlas [11], and it is generally acknowledged that gene regulatory networks (GRNs) orchestrate fate-decision procedures. Namely, conserved regulatory programs further bring us a conserved topology of core GRNs. Thus, the logics of regulation, as another vital element in GRNs, is naturally under the spot light (related to the introduction, lines 99-120 of the revised manuscript). Nevertheless, to our knowledge, regulatory logic in cell fate decisions has received only scant attention. We hope that our elucidation of the role of logic motifs in cell fate decisions will attract more inquiries in community into GRN’s regulatory logic.

**Public reviews**

In this manuscript, Xue and colleagues investigate the fundamental aspects of cellular fate decisions and differentiation, focusing on the dynamic behaviour of gene regulatory networks. It explores the debate between static (noise-driven) and dynamic (signal-driven) perspectives within Waddington's epigenetic landscape, highlighting the essential role of gene regulatory networks in this process. The authors propose an integrated analysis of fate-decision modes and gene regulatory networks, using the Cross-Inhibition with Self-activation (CIS) network as a model. Through mathematical modelling, they differentiate two logic modes and their effect on cell fate decisions: requires both the presence of an activator and absence of a repressor (AA configuration) with one where transcription occurs as long the repressor is not the only species on the promoter (OO configuration).

The authors establish a relationship between noise profiles, logic-motifs, and fate-decision modes, showing that defining any two of these properties allows the inference of the third. They also identify, under the signal-driven mode, two fundamental patterns of cell fate decisions: either prioritising progression or accuracy in the differentiation process. The authors apply this analysis to available high-throughput datasets of cell fate decisions in hematopoiesis and embryogenesis, proposing the underlying driving force in each case and utilising the observed noise patterns to nominate key regulators.

The paper makes a substantial contribution by rigorously evaluating assumptions in gene regulatory network modelling. Notably, it extensively compares two model configurations based on different integration logic, illuminating the consequences of these assumptions in a clear, understandable manner. The practical simulation results effectively bridge theoretical models with real biological systems, adding relevance to the study's insights. With its potential to enhance our understanding of gene regulatory networks across biological processes, the paper holds promise. Its implications extend practically to synthetic circuit design, impacting biotechnology. The conclusions stand out, addressing cell fate decisions and noise's role in gene networks, contributing significantly to our understanding. Moreover, the adaptable approach proposed offers versatility for broader applications in diverse scenarios, solidifying its relevance beyond its current scope.

We thank the reviewers for their enthusiasm for our work, and appreciate the professional, insightful and encouraging assessment.

However, the manuscript in its current form also has some important weaknesses, including the lack of clarity in the text and the questionable generality of specific observations.

We thank the reviewers for this comment. We have reviewed the manuscript and made the necessary adjustments to improve its clarity. We do hope that this revision meets the reviewers’ expectations on the clarity and comprehensiveness of our analysis.

For instance, even when focusing on the CIS network, the effect of alternative model implementations is not discussed. Notably, the input signals are only considered as an additive effect over the differential equations, while signals can potentially affect each of the individual processes.

We agree with the reviewers’ comment that signals may affect at each level of the central dogma, including transcription, translation, etc. Further, we have also included additional section titled “limitation of this study” on this point in the revised manuscript, and explicitly point to the potential limitations of our models. Please refer to Page 25 of the revised manuscript, lines 769-771 (see below).

“In addition, for simplicity and intuition, we here considered signals as uncoupled and additive effects in ODE models, due to feasible mapping in real biological systems, such as ectopic overexpression.”

The proposed model allows for a continuum of interactions/competition between transcription factors, yet only very restrictive scenarios are explored (strict AND/OR logic operations).

We thank the reviewers for this comment, and appreciate them sharing the potential for further generalization of our framework. Indeed, in addition to logic operations, our framework is able to be applied to all two-node circuits (3^4=81 in total), including mutual activation with self-activation. As the focus of this work is to illustrate the role of logic motifs in cell fate decisions, we mainly concentrated on two classical, intuitive and representative (at least to us) logic operations AND/OR in the context of the CIS network. Nonetheless, we already have four combinations to consider (two logic motifs and two driving forces). And we feel that the currently involved scenarios have properly fulfilled our need to manifest the role of logic motifs. Hence, we carefully decided not to further explore more logic operations in this work. Instead, we have included additional section titled “limitation of this study” in the revised manuscript. Please refer to Page 25 of the revised manuscript, lines 760-762.

“Although our framework enables the investigation of more logic motifs, we chose two classical and symmetrical logic combinations for our analysis. Future work should involve more logic gates like XOR and explore asymmetrical logic motifs like AND-OR.”

Moreover, how the model parameters are chosen throughout the paper is not clear. Similarly, the concentration and times are not clearly specified, making their comparison to experimental data troublesome.

We thank the reviewers for this comment. Regarding how to specify parameters in our model, we have now revised the manuscript. Please refer to Page 5 of the revised manuscript, lines 179-181 (“Benchmarking the Boolean models with different logic motifs (Fig2.B; see Methods), we reproduced the geometry of the attractor basin in the continuous models resembling those represented by corresponding Boolean models (Fig2.C; see Methods).”). In terms of concentration and time, we acknowledge that their units are arbitrary compared to a real experimental system. We now have noted this point in the legend of corresponding figures (Fig2.C, Fig3.B&D, Fig6.B-C, Fig7.E).

We would like to highlight that our entire work is organized in a model-driven fashion (also called top-down). We did not fine-tune the sets of parameters used in our model to specifically match the experimental data. Actually, it is also a longstanding challenge in computational biology since experimental datasets are usually insufficient to specify the parameters in a dynamical model. So, in general, it is inevitable to involve more assumptions such as non-Markov process[12, 13] and may lead to artifacts. Thus, we decided to draw qualitative conclusions (e.g., trends over time) from a quantitative model with sampling of parameter sets. Hence, we did not intentionally tailor our models to fit different datasets (i.e., all models used in our work share same basic setting of parameters), mapping into real biological systems in a top-down manner.

Regarding clarity, how the general model (equations 1-2) transforms into the specific cases evaluated in the paper is not clearly stated in the main text, nor are the positive and negative effects of individual transcription factors adequately explained. Similarly, in the main text and Figure 2, the authors refer to a Boolean model. However, they do not clearly explain how this relates to the differential equation model, nor its relevance to understanding the paper.

We thank the reviewers for this comment, as it has prompted us to better clarify our manuscript. We have adjusted the manuscript accordingly and made the necessary adjustments to improve its clarity.

Additionally, the term "noise levels" is generally used to refer to noise introduced in the "noise-driven" analysis (i.e., as an input or parameter in the models). Nonetheless, it is later claimed to be evaluated as an intrinsic property of the network (likely referring to expression level variability measured by the coefficient of variation).

We greatly appreciate the reviewers pointing this ambiguity out. The term of “noise level” was indeed used to refer the strength of the noise in the models in Section 1-4. For classifying different logic motifs with two driving forces, we needed a practical metric that can be quantified from data, and we found population-level gene expression variance (i.e., “noise level” in line 398) is useful which defined as the coefficient of variation.

For clarity, we carefully decide to substitute “expression variance” for “noise level” presented in Section 5-6. We have amended the manuscript accordingly.

Finally, some jargon is introduced without sufficient context about its meaning (e.g., "temporal fully-connected stage").

Regarding the jargon of "temporal fully-connected stage", we have realized that this term was slightly vague and in need of improvement. Instead, we now employ “transitory fully-connected stage” in the revised manuscript to underline the short emergence of this particular stage. Please refer to Page 10-11 of the revised manuscript, lines 316-327 (see below).

“Notably, in the AND-AND motif we observed a brief intermediated stage before S attractor disappears, where all three fates are directly interconnected (Fig4.C 2nd panel and D 2nd panel, Fig.4E). To manifest the generality, we globally screened 6,213 groups of parameter sets under the AND-AND motif, and this logic-dependent intermediated stage can be observed for 82.7% of them (see Methods; Table S1), indicating little dependence on particular parameter setting (1.8% in the OR-OR motif). Unlike the indirect attractor adjacency structure mediated by S attractor (Fig2.D), the solution landscape with fully-connected structure facilitates transitions between any two pairs of fates. Furthermore, this transitory fully-connected stage locates between the fate-undetermined stage (Fig4.C top panel) and fate-determined stage (Fig4.C 3rd panel), comparable to the initiation (or activation) stage before the lineage commitment in experimental observations [5-7]. Therefore, we suspected that the robust fully-connected stage in the AND-AND motif may correspond to a specific period in cell fate decisions.”

Additionally, proper discussion of previous work is also missing. For instance, the dynamics of the CIS network investigated by the authors have been extensively characterised (see e.g., Huang et al., Dev Biol, 2007), and how the author's results compare to this previous work should be discussed. In particular, the central assumptions behind the derivation of the model proposed in the manuscript must be assessed in the context of previous work.

Thanks for pointing this out. We have extended the discussion to include above points. We have also discussed and cited the work of Huang mentioned above. Please refer to Page 22, lines 644-647 in the revised manuscript (see below).

“One of the most representative work is that Huang et al. [14] modeled the bifurcation in hematopoiesis to reveal the lineage commitment quantitatively. Compared to simply modularizing activation or inhibition effect by employing Hill function in previous work, our models reconsidered the multiple regulations from the level of TF-CRE binding.”

References

(1) Ackers, G.K., A.D. Johnson, and M.A. Shea, Quantitative model for gene regulation by lambda phage repressor. Proc Natl Acad Sci U S A, 1982. 79(4): p. 1129.

(2) Shea, M.A. and G.K. Ackers, The OR control system of bacteriophage lambda: A physical-chemical model for gene regulation. Journal of Molecular Biology, 1985. 181(2): p. 211-230.

(3) Hunziker, A., et al., Genetic flexibility of regulatory networks. Proc Natl Acad Sci U S A, 2010. 107(29): p. 12998-3003.

(4) Kittisopikul, M. and G.M. Suel, Biological role of noise encoded in a genetic network motif. Proc Natl Acad Sci U S A, 2010. 107(30): p. 13300-5.

(5) Brand, M. and E. Morrissey, Single-cell fate decisions of bipotential hematopoietic progenitors. Curr Opin Hematol, 2020. 27(4): p. 232-240.

(6) Zhang, Y., et al., Hematopoietic Hierarchy - An Updated Roadmap. Trends Cell Biol, 2018. 28(12): p. 976-986.

(7) Arinobu, Y., et al., Reciprocal activation of GATA-1 and PU.1 marks initial specification of hematopoietic stem cells into myeloerythroid and myelolymphoid lineages. Cell Stem Cell, 2007. 1(4): p. 416-27.

(8)Kamimoto, K., et al., Dissecting cell identity via network inference and in silico gene perturbation. Nature, 2023. 614(7949): p. 742-751.

(9) Hammelman, J., et al., Ranking reprogramming factors for cell differentiation. Nat Methods, 2022. 19(7): p. 812-822.

(10) Semrau, S., et al., Dynamics of lineage commitment revealed by single-cell transcriptomics of differentiating embryonic stem cells. Nat Commun, 2017. 8(1): p. 1096.

(11) Li, J., et al., Deep learning of cross-species single-cell landscapes identifies conserved regulatory programs underlying cell types. Nature Genetics, 2022. 54(11): p. 1711-1720.

(12) Stumpf, P.S., F. Arai, and B.D. MacArthur, Modeling Stem Cell Fates using Non-Markov Processes. Cell Stem Cell, 2021. 28(2): p. 187-190.

(13) Stumpf, P.S., et al., Stem Cell Differentiation as a Non-Markov Stochastic Process. Cell Syst, 2017. 5(3): p. 268-282 e7.

(14) Huang, S., et al., Bifurcation dynamics in lineage-commitment in bipotent progenitor cells. Dev Biol, 2007. 305(2): p. 695-713.
